# One‐Step Soaking Strategy toward Anti‐Swelling Hydrogels with a Stiff “Armor”

**DOI:** 10.1002/advs.202206242

**Published:** 2023-01-22

**Authors:** Xueyu Dou, Hufei Wang, Fei Yang, Hong Shen, Xing Wang, Decheng Wu

**Affiliations:** ^1^ College of Chemistry Chemical Engineering and Materials Science Key Laboratory of Molecular and Nano Probes Ministry of Education Collaborative Innovation Center of Functionalized Probes for Chemical Imaging in Universities of Shandong Institute of Molecular and Nano Science Shandong Normal University Jinan 250014 China; ^2^ Beijing National Laboratory for Molecular Sciences Institute of Chemistry Chinese Academy of Sciences Beijing 100190 China; ^3^ University of Chinese Academy of Sciences Beijing 100049 China; ^4^ Department of Biomedical Engineering Southern University of Science and Technology Shenzhen 518055 China

**Keywords:** anti‐swelling, core/shell structure, double‐network hydrogels, ionic coordination, soaking strategy

## Abstract

Double‐network (DN) hydrogels consisting of noncovalent interacting networks are highly desired due to their well‐controlled compositions and environmental friendliness, but the low water resistance always impairs their mechanical strength. Here, an anti‐swelling hydrogel possessing the core/shell architecture through rational regulation of multiple weak noncovalent interactions is prepared. A composite hydrogel consists of chitosan (CS) and poly(*N*‐acryloyl 2‐glycine) (PACG), readily forming the shell‐structured DN hydrogel after soaking in a FeCl_3_ solution because of in situ formation of chain entanglements, hydrogen bonds, and ionic coordination. The produced DN hydrogels exhibit excellent anti‐swelling behaviors and mechanical durability for over half a year, even in some strict situations. Taking the merits of noncovalent bonds in adjustability and reversibility, the swelling property of these hydrogels can be easily customized through control of the ion species and concentrations. A dynamically reversible transition from super‐swelling to anti‐swelling is realized by breaking up and rebuilding the metal‐coordination complexes. This facile but efficient strategy of turning the noncovalent interactions and consequently the mechanics and anti‐swelling properties is imperative to achieve the rational design of high‐performance hydrogels with specific usage requirements and expand their applicability to a higher stage.

## Graphical abstract

A soaking strategy is reported to produce a class of tough and anti‐swelling double‐network hydrogels, possessing a dense polymer layer as the protective “armor” through elaborate regulation of noncovalent interactions and thereby the aggregation of polymers. Owing to the dynamic and configurable nature of noncovalent bonds, customized mechanics and swelling are in situ accessed, which endows them adaptive nature applied for different situations.

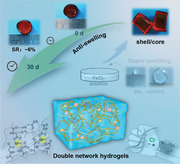

## 1 Introduction

Hydrogels, three‐dimensional (3D) polymeric network systems with similar physical properties to natural tissue, have been considered to be one of the best candidates as “soft and wet” materials for wide applications in biomedical fields, such as drug delivery,^[^ ^1^, ^2^, ^3^ ^]^ tissue engineering^[^ ^4^, ^5^, ^6^, ^7^ ^]^ and soft electronics.^[^ ^8^, ^9^, ^10^, ^11^ ^]^ In recent years, much effort has been devoted to developing high‐strength hydrogels due to their great potential for extended usage in persistent load‐bearing applications, including double networks,^[^ ^12^, ^13^, ^14^, ^15^, ^16^ ^]^ topological sliding networks,^[^ ^17^, ^18^, ^19^ ^]^ and composite reinforcement mechanisms.^[^ ^20^, ^21^, ^22^ ^]^ Nonetheless, owing to the hydrophilic crosslinked network, as‐prepared hydrogels always involve inevitable swelling in aqueous environments,^[^ ^23^, ^24^, ^25^ ^]^ which suffers from a sharp decline in mechanical strength that is undesired in many situations, particularly when applied in vivo, such as artificial tissues or implantable electronics.^[^ ^10^, ^26^, ^27^ ^]^ To address such issues, diverse approaches have been utilized to produce swelling‐resistant hydrogels through subtle regulation of the hydrophilic‐lipophilic balance,^[^ ^28^, ^29^ ^]^ crosslinking density^[^ ^30^, ^31^, ^32^ ^]^ as well as contents and components of the monomers.^[^ ^33^, ^34^ ^]^ However, such hydrogels are often prepared involving in sophisticated recipes. In addition, these methods are unable to dynamically tune hydrogel swelling in situ because of pre‐setting functionality and proportion.

Noncovalent bonds (e.g., hydrogen bonds and ionic bonding) are commonly weak yet prominent interactions in biological systems and material science.^[^ ^35^, ^36^, ^37^ ^]^ Recent years have witnessed rapid advances in the construction of physical hydrogels to obtain superior properties (e.g., external stimuli‐responsiveness,^[^ ^38^, ^39^, ^40^, ^41^ ^]^ and self‐healing^[^ ^42^, ^43^ ^]^) due to the dynamic and configurable nature of noncovalent bonds. In nature, the hard exoskeleton of many creatures, such as chrysomallon squamiferum^[^ ^44^, ^45^ ^]^ called the “iron man” of the animal kingdom is held together by noncovalent interactions between chitin (nitrogenous polysaccharide compounds), arthropodin and inorganic compounds, and these advanced self‐assembled structures contribute to internal protection and support for their soft bodies. Inspired by biological bionics in nature, we expect to create a shell layer based on strong noncovalent interactions like the “armor” that can protect the dimensional stability of hydrogels.

Herein, we reported a simple and universal soaking strategy to produce a class of anti‐swelling hydrogels with a protective “armor” by virtue of rational regulation of noncovalent interactions and thereby the conformation of polymers. Chitosan/poly(*N*‐acryloyl 2‐glycine) (CS/PACG) composites were used as a model system here, because of their well‐controlled structures in different mediums (Scheme S1, Figures S1 and S2, Supporting Information). The composite hydrogel was prepared by ultraviolet (UV) triggered polymerization of *N*‐acryloyl 2‐glycine (termed as ACG) containing a crosslinker, *N*,*N* *′*‐methylene‐bis‐acrylamide (MBA) with an embedded short‐chain chitosan, and then converted to stiff and tough double‐network (DN) hydrogels after soaking in FeCl_3_ solution to conduct the conversion of polymer aggregations. Wherein, the Cl^−^ and Fe^3+^ in ferric chloride can interact with amino groups of chitosan and carboxyl groups of ACG to form chain‐entanglement and ionic crosslinked networks, respectively. Owing to the slow diffusion of ferric chloride from the gel surface to the inside, a gradient distribution of carboxyl‐Fe^3+^ coordination facilitated to yield the DN hydrogels with a soft core/stiff shell structure. The resultant hydrogels exhibited well‐maintained network structure, and tenable mechanical property even after swelling in water for more than half a year. Tuning the various ion concentrations could facilely modulate the dense layer to subsequently tailor the hydrogel's mechanics and swelling properties over a large window (e.g., compressive modulus from 0.17 to 4.25 MPa, the swelling ratio from −45% to 44 000%). Furthermore, we demonstrated that the control of the soaking order of different metal ions solutions could enable a reversible “super‐anti” swelling transformation as needed. This strategy synergistically combines a double network toughening mechanism and bioinspired material design concept to construct a kind of novel swelling‐resistant hydrogel by means of a dense outer layer. Therefore, we believe that such a simple and effective strategy will provide a new perspective to build tough anti‐swelling hydrogels for broader applications.

## 2 Results and Discussion

### 2.1 Design and Preparation of Shell‐Structured DN Hydrogels

**Figure 1:**
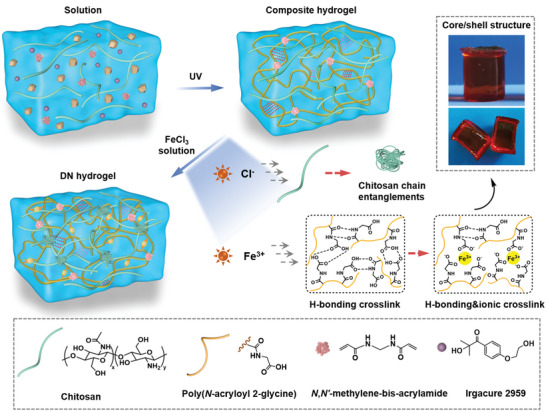
Construction of shell‐structured CS/PACG DN hydrogels. One‐step soaking to regulate dual networks for preparation of CS/PACG DN hydrogels and schematics of the multiple interactions between polymer chains and various ions.

The formation of CS/PACG DN hydrogels is depicted in **Figure** **1**. A CS/PACG composite hydrogel was first prepared via UV‐initiated radical polymerization in which the PACG was cross‐linked by covalent bonds/dual hydrogen bonds and CS was well‐dispersed in the hydrogel matrix. Subsequently, the composite hydrogel was soaked into an aqueous FeCl_3_ solution to produce a CS/PACG‐Fe^3+^ DN hydrogel. In this process, chloride and ferric ions were permeated into the hydrogel and served as cross‐linkers to form amino‐anion and carboxyl‐cations domains: i) the chloride salt triggered the salting‐out of polymers, thereby resulting in the spontaneous collapse of chitosan chains to form the chain‐entanglement network^[^ ^15^ ^]^; and ii) the strong carboxyl/Fe^3+^ electrostatic interaction induced ionic crosslinks of PACG.^[^ ^46^, ^47^ ^]^

Three composite hydrogels designated as CS_x_/PACG were prepared, where x represented the original weight percentage of chitosan (0.8, 1.6, and 3.9) in the hydrogels (Figures S3–S5, Supporting Information). Remarkably, the composite hydrogels exhibited better mechanical properties than the PACG single network (SN) hydrogel (Figures S6–S8, Supporting Information). However, as CS concentration increased from 0.8 to 3.9 wt.%, the ultimate stress and strain were decreased. Toughness and modulus exhibited similar trends with a decrease from 1.63 to 0.30 MJ m^−3^ and 0.07 to 0.04 MPa, respectively due to the looser micropores (Figure S9, Supporting Information). Considering the structure together with mechanics, the CS_1.6_/PACG hydrogel was chosen as a model system for further study.

**Figure 2:**
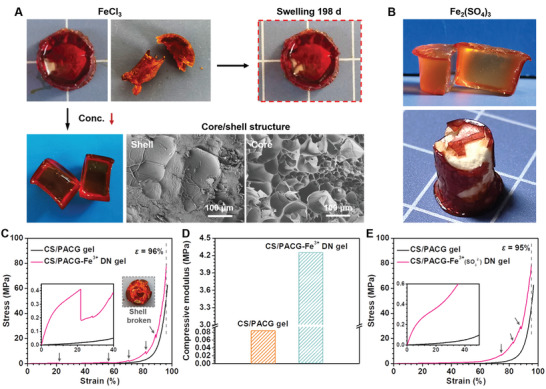
Structure and mechanical properties of the CS/PACG hydrogels treated with ferric ion. A) Photographs and SEM images of the hydrogels with different soaking conditions: 1.5 m FeCl_3_ solution of 6 h (upper) and 0.1 m FeCl_3_ solution of 1 h (lower). B) Photographs showing the core/shell structure of the hydrogel after soaking in 0.05 m Fe_2_(SO_4_)_3_ solution for 1 h. C) Compressive stress‐strain curves (inset shows the destruction of shell after compression) and D) corresponding modulus of the hydrogels. E) Compressive stress‐strain curves of the CS/PACG‐Fe^3+^(SO_4_^2−^) DN hydrogel.

When the composite hydrogel was soaked in a 1.5 m FeCl_3_ solution, a solid‐like CS/PACG‐Fe^3+^ DN hydrogel with excellent water‐resistant ability was produced, which could be attributed to powerful carboxyl‐Fe^3+^ interaction (**Figure** **2**A; Figures S10 and S11, Supporting Information). To validate the above assumption, we evaluated the effects of salt concentrations on the structure of hydrogels (Figure S12, Supporting Information). Expectedly, instead of forming the dense bulk, a gradient gel was yielded as the concentration decreased to 0.1 m. The resulted DN hydrogel had two layers with a soft core surrounded by a stiffer shell (Figure 2A; Figure S13, Supporting Information). The thickness of the outer layer could be well customed by tuning soaking concentration and time (Figures S12, S14, and S15, Supporting Information). Such a distinct structure was also observed when the composite hydrogel was soaked in Fe_2_(SO_4_)_3_ solution, thus indicating the main driving forces for the formation of the stiff shell originated from the strong metal coordination (Figure 2B). The Fe^3+^ diffused from the surrounding solution into the gel matrix and interacted with the carboxyl group. A layer of dense cross‐linked polymer network thus formed and grew in a radial direction inward from the surface because of the gradient diffusion behavior (Figure S16, Supporting Information). Different crosslinking density was confirmed by the microstructure in scanning electron microscopy (SEM) images and the changes of characteristic absorption peak (C≐O in the carboxyl group) in fourier transform infrared (FTIR) results (Figure 2A; Figures S17 and S18, Supporting Information). Unless otherwise noted, the CS/PACG‐Fe^3+^ represents the DN hydrogel treated with FeCl_3_.

### 2.2 Mechanical and Swelling Properties of Shell‐Structured DN Hydrogels

The distinct network architecture endowed the CS/PACG‐Fe^3+^ (taking Cl^−^ or SO_4_^2−^ as anion) DN hydrogels with outstanding mechanical properties. The compressive strength significantly increased to 78.5 MPa for the CS/PACG‐Fe^3+^(Cl^−^) DN hydrogel compared to 52.4 MPa of the composite hydrogel at a strain of 96% (Figure 2C). In addition, the compressive modulus reached 4.25 MPa, which was ≈51 times higher than that of the pristine hydrogel (Figure 2D). A similar trend was also recorded for the CS/PACG‐Fe^3+^(SO_4_^2−^) DN hydrogel (Figure 2E; Figure S19, Supporting Information). The stiff shell greatly improved the mechanical strength of hydrogel but limited their elasticity natures, thereby that CS/PACG‐Fe^3+^(Cl^−^) and CS/PACG‐Fe^3+^(SO_4_^2−^) DN hydrogels cannot be stretched as the conventional gels did (Figures S20, Supporting Information). The rupture of the shell under large deformation was confirmed by wobbly points in the compression curve.

**Figure 3:**
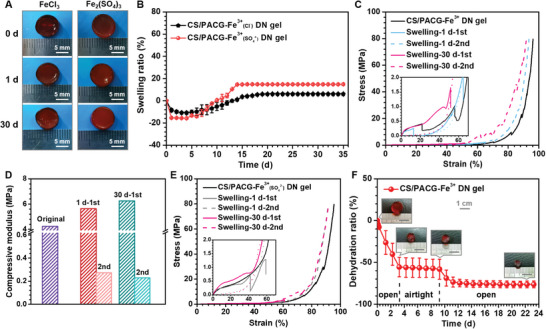
Anti‐swelling performance of the CS/PACG hydrogels treated with ferric ion. A) Optical images and B) anti‐swelling curves of the CS/PACG‐Fe^3+^(Cl^−^) and CS/PACG‐Fe^3+^(SO_4_^2−^) DN hydrogels in water. Compressive performance of C,D) the CS/PACG‐Fe^3+^(Cl^−^) and (E) the CS/PACG‐Fe^3+^(SO_4_^2−^) DN hydrogels with different swelling time in water. F) Dehydration behavior of the hydrogels in open or airtight system.

In view of this denser blocking layer, the CS/PACG‐Fe^3+^ DN hydrogel can maintain structural and mechanical stability even after swelling for one month (**Figure** **3**A,B; Figures S21 and S22, Supporting Information). Minimal volume expansion was observed with a low equilibrium swelling ratio of 5.8% and 14.8% for the CS/PACG‐Fe^3+^(Cl^−^) and CS/PACG‐Fe^3+^(SO_4_^2−^), respectively. Notably, both the initial modulus and strength were enhanced with the increase of swelling time (Figure 3C‐E; Figure S23, Supporting Information). The compressive stress of the CS/PACG‐Fe^3+^(Cl^−^) and CS/PACG‐Fe^3+^(SO_4_^2−^) hydrogels increased from 24.8 to 43.8 MPa and 36.5 to 62.0 MPa at a swelling for one day (1 d), which further increased to 63.9 MPa and 67.7 MPa with the time extending to 30 d at a strain of 90%, respectively. Additionally, the compressive modulus of the CS/PACG‐Fe^3+^(Cl^−^) and CS/PACG‐Fe^3+^(SO_4_^2−^) hydrogels were also enhanced to 6.28 MPa and 5.14 MPa after swelling for 30 d, respectively, compared to 4.25 MPa and 3.35 MPa of their original hydrogels. This strengthening behavior could be attributed to the gradual reconstitution of Fe^3+^ ions and structural optimization of the cross‐linking network. We also noticed a sharp decline in modulus under the immediate second compression, which was correlated with the gradient structure. Moreover, it is worth noting that the produced hydrogels exhibited superior anti‐swelling ability in deionized water at 37 °C and 0.9 g mL^−1^ NaCl solution, different pH solutions at room temperature (Figure S24, Supporting Information). However, they underwent large swelling because of the weakened hydrogen bonding/ionic‐based cross‐linking in basic situations. For example, the swelling ratio of CS/PACG‐Fe^3+^ was hard to be measured at 4 d, because of difficulty in maintaining integrity in alkaline medium. In addition, the CS/PACG‐Fe^3+^ DN hydrogel began to dehydrate and shrink upon exposure to air, yet significantly slower than that of the conventional hydrogels (Figure 3F). Particularly, the dehydration behavior can be almost interrupted by placing the gel in the airtight condition and restarted by resetting it in the air. This indicated that the hydrogel could be conveniently stored for use whenever needed.

### 2.3 Tunable Mechanical Properties and Swelling Behaviors via Adjustment of Metal Cations

**Figure 4:**
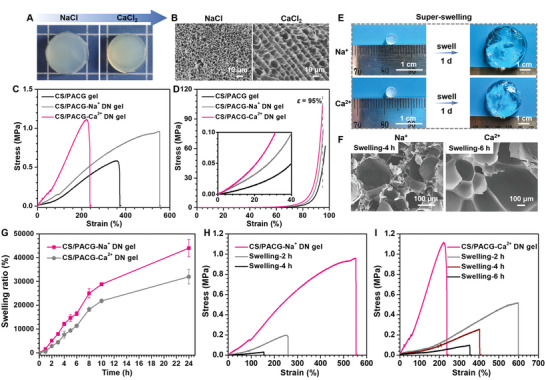
Tunable mechanical properties and swelling behaviors of the CS/PACG hydrogels by various metal cations. A) Photographs and B) SEM images of the CS/PACG hydrogels after soaking in 1.5 m NaCl and CaCl_2_ solutions for 6 h. C) Tensile and D) compressive stress‐strain curves of the CS/PACG hydrogels soaked in chloride salts. E) Diagram and F) SEM images showing the extent of swelling after 4 h, 6 h, and 1 d. G) Swelling curves of the CS/PACG‐Na^+^ and CS/PACG‐Ca^2+^ hydrogels in deionized water. Mechanical properties of the (H) CS/PACG‐Na^+^ and (I) CS/PACG‐Ca^2+^ hydrogels with different swelling time in water.

To better understand the effect of cations, we prepared two control hydrogels treated with other chloride salts. As depicted in **Figure** **4**A; Figure S25 (Supporting Information), the translucent or opaque CS/PACG‐Na^+^ and CS/PACG‐Ca^2+^ DN hydrogels were obtained. Instead of the core/shell structure, typical homogeneous honeycomb pores were formed, suggesting the weak aggregation states in the presence of monovalent or divalent cations (Figure 4B). Compared to the CS/PACG‐Fe^3+^ hydrogel, the CS/PACG‐Na^+^ and CS/PACG‐Ca^2+^ DN hydrogels exhibited excellent elasticity and toughness. Both of them could withstand various deformations of compression, stretching, knotting, and twisting without any noticeable deformation (Figure S26, Supporting Information). Upon removal of the pressure, the hydrogels could quickly recover to their original shapes, indicating the excellent shape‐recovery property. Due to the excellent biocompatibility and adaptable gel‐forming ability using this simple one‐step soaking method, the CS/PACG DN hydrogels can be readily adapted to different complex shapes, such as branchs, rabbits, and starlight sticks. This suggested that the hydrogel can be applied as suitable 3D scaffolds for tissue engineering.

The typical stress‐strain behaviors were further performed to quantitatively examine the mechanical properties of the CS/PACG‐Na^+^/Ca^2+^ DN hydrogels. Similarly, the higher strength and stiffness than the composite hydrogel were recorded (Figure 4C,D; Figures S27 and S28, Supporting Information). The CS/PACG‐Na^+^ and CS/PACG‐Ca^2+^ hydrogels gave a stress of 0.96 and 1.12 MPa, and modulus of 0.14 and 0.36 MPa, respectively. The compressive strength of the CS/PACG‐Ca^2+^ hydrogel was significantly enhanced to be ≈103.6 MPa, which surpassed the values reported in most previous works about tough hydrogels. Note that it was not possible to reach the preset strain (*ε*_set_ = 97%) due to the limitations of the tensile tester. The CS/PACG‐Na^+^ hydrogel exhibited a similar trend in compressive strength from 45.1 to 79.7 MPa at the strain of ≈95%. Owing to the synergistic effect of multiple physical interactions, including chain‐entanglement, ionic networks, and hydrogen bonding, these DN hydrogels exhibited good energy dissipation mechanism and self‐recovery with the verification of a large hysteresis loop and short recovery time (Figures S29–S31, Supporting Information).

Similar to conventional hydrogels, the CS/PACG‐Na^+^/Ca^2+^ DN hydrogels showed a swelling‐weakening behavior, which suffered from a sharp decline in mechanical strength after the expansion due to the dilution of network. The swelling ratio of CS/PACG‐Na^+^ and CS/PACG‐Ca^2+^ hydrogels increased by ≈5110% and ≈2820% after immersion in deionized water for two hours (2 h), which further increased to ≈44 000% and ≈32 000% with the time extending to 24 h (Figure 4E‐G; Figure S32, Supporting Information). If they were soaked for a longer time, the swelling ratio was hard to be measured because of the difficulty in maintaining their integrity. Remarkably, the ultimate stress decreased significantly from 0.96 to 0.03 MPa for the CS/PACG‐Na^+^ hydrogel and 1.11 to 0.26 MPa for the CS/PACG‐Ca^2+^ hydrogel after 4 h, respectively (Figure 4H,I; Figure S33, Supporting Information). The compressive stress also exhibited a decreased trend from 79.8 to 26.5 MPa and 104.0 to 22.4 MPa (Figures S34 and S35, Supporting Information). The larger pore size and looser structure observed in SEM well agreed with the above results (Figure 4F; Figure S36, Supporting Information).

### 2.4 A Universal Approach to Building Shell‐Structured DN Hydrogels with Anti‐Swelling Property

**Figure 5:**
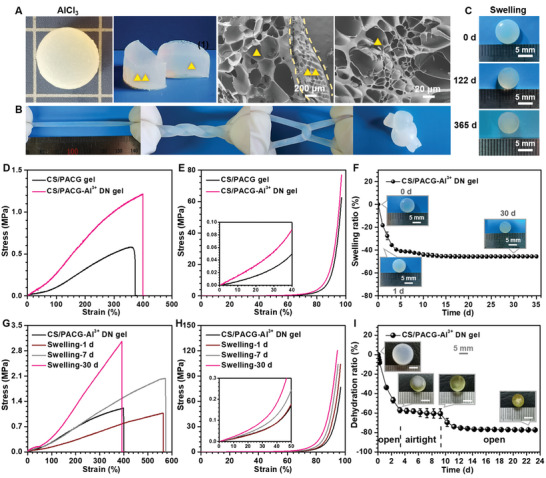
The generality of stiff and anti‐swelling hydrogels produced by soaking strategy. A) Photographs and SEM images of the CS/PACG hydrogels after soaking in 0.1 m AlCl_3_ solution for 1 h. B) The extraordinary mechanical properties of the CS/PACG‐Al^3+^ hydrogel: stretching, twisting, crossover stretching, and knotting. C) Photographs of the CS/PACG‐Al^3+^ hydrogel after swelling of 1, 122, and 365 d. D) Tensile and E) compressive stress‐strain curves of the CS/PACG and CS/PACG‐Al^3+^ hydrogel. F) Swelling curves, G) tensile and H) compressive performance of the CS/PACG‐Al^3+^ hydrogel with predetermined swelling time in water. I) Dehydration behavior of the hydrogel in open or airtight system.

Besides for the trivalent cation of Fe^3+^, we also fabricated the CS/PACG‐Al^3+^ hydrogel using the same soaking strategy in AlCl_3_ solutions. Interestingly, adjusting the multivalent cations from Fe^3+^ to Al^3+^ did not yield a stiff shell, and instead formed the flexible dense layer that could be forcefully pulled along with hydrogel interior without failure, even if it was twisted to a helical shape or tied to a knot (**Figure** **5**A,B). This difference was rationally linked to the cross‐linking degree between the PACG and cross‐linker ions. Compared to Fe^3+^ cations with larger ion radius (0.645 Å), Al^3+^ cations with smaller ion radius (0.535 Å) formed a looser structure,^[^ ^48^ ^]^ thus resulting in a lower cross‐linked arrangement. In addition, the Al^3+^ formed a mixture of bi‐, or tridentates with carboxyl groups, which caused a weaker coordination than that of Fe^3+^.^[^ ^49^, ^50^ ^]^ Even so, the CS/PACG‐Al^3+^ hydrogel still exhibited excellent mechanical properties, good self‐recovery, convenient storage, and outstanding anti‐swelling performance, which could retain its initial pore structure and mechanical strength after one year even in some strict situations (Figure 5C–H; and Figures S37–S43, Supporting Information). Similar to the CS/PACG‐Fe^3+^ hydrogel, the CS/PACG‐Al^3+^ hydrogel also showed controllable water retention performance (Figure 5I).

Based on the above results, it is rational to predominantly ascribe this intriguing core/shell gradient structure to strong carboxyl‐Fe^3+^ coordination. A three‐dimensional valent bonding structure ((COO^−^)_3_ M)) (M refers to trivalent cations) forms as the Fe^3+^ ions diffuse from the surrounding solution into the hydrogel and hold with the carboxyl group, thus resulting in a dense polymeric network. Owing to the gradient concentration of ferric iron, an outside‐in growth is proceeded to form a hydrogel with two distinct layers that of a soft core surrounded by a stiff shell. Such a high crosslinking polymer layer endowed the hydrogel with excellent dimensional stability. When the ionic interactions were weakened, this dense layer changed from a stiff one to a flexible one or even disappear, achieving the transformation from anti‐swelling to super‐swelling hydrogels. The cross‐linker ions (i.e., charge and ion radius of multivalent cations) determined the cross‐linking degree of network and properties (i.e., swelling and mechanics) of hydrogels. We proposed that cations with the higher charge and larger ion radius will interact with more carboxylic groups of different PACG chains at the same time to generate a larger coordination number and a tighter structure, which may also be applied to construct the swelling‐resistant hydrogels with protective shell layers.

### 2.5 Reversible Transition from “Super‐ to Anti‐” Swelling

**Figure 6:**
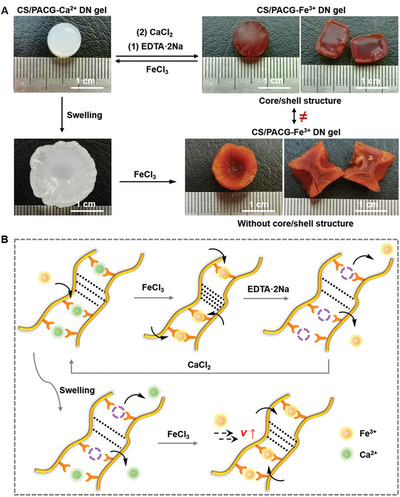
Reversible transition from “super‐ to anti‐” swelling. A) Photographs and B) schematic diagram of reversible transformation between “super‐anti” swelling state upon immersing into the various metal ion solutions.

Owing to the dynamic nature of metal‐coordination bonds, we also found that the CS/PACG‐Ca^2+^ hydrogel transformed into a core/shell one accompanied with the ion exchange operation to replace Ca^2+^ with Fe^3+^ in situ (**Figure** **6**). This was because Fe^3+^ cations with higher charge and larger ion radius could preferentially fill a larger space between the blocks of PACG polymers to form a tighter structure compared with Ca^2+^. Reversibly, these hydrogels could also be converted to the original ones by sequentially incubating them in ethylenediaminetetraacetic acid disodium salt (EDTA·2Na), a competitive molecule that can break up the carboxyl‐metal coordination, and then immersing in an aqueous CaCl_2_ solution to reform carboxyl‐Ca^2+^ complexes. It is notable that when placed the swollen one into the FeCl_3_ solution, instead of following gradient hydrogel, the hydrogel almost recovered to their original volume yet homogeneous structure, which was attributed to the more and more Fe^3+^ ions could facilitate to permeate into the swelling hydrogel and evenly disperse to react with the carboxyl groups of PACG. Whether this transformation is completely reversible (e.g., microstructure, mechanical strength) and the transformation hysteresis effects will be systematically given in our following work.

## 3 Conclusion

In summary, we fabricated a robust CS/PACG DN hydrogel with an “armor” using a facile and universal soaking strategy. Taking advantage of multiple physical interactions like chloride ion‐assisted chitosan chains‐entanglements, ferric iron‐induced ionic networks, and hydrogen‐bond interactions, the resultant DN hydrogels exhibited the intact networks and excellent mechanical property with high strength and fracture energy that surpassed the most reported tough hydrogels. Central to the excellent mechanics nature of hydrogel is the formation of a stiff shell structure by strong carboxyl‐Fe^3+^ coordination interactions, which also served as a “protective armor” to retain dimensional stability and anti‐swelling ability even after swelling in water for over half a year. Once weakening the interaction between carboxyl‐low valence metal‐coordination complexes upon switching to the low metal ion solutions, the CS/PACG composite hydrogels could convert into super‐swelling DN hydrogels with a maximal swelling ratio of 44 000%. Note that simple control of soaking order of various metal ions solutions could enable a reversible “super‐swelling”⇔“anti‐swelling” transformation, which holds considerable promise to improve the applicability of hydrogels. Considering the widespread existence of metal‐coordination effects in various polymer systems, we are convinced that the proposed strategy is universal and applicable to produce natural or synthetic hydrogels with expanded functionalities in biomedicine, robotics, and other manufacturing fields.

## 4 Experimental Section

### Materials

Short‐chain chitosan (CS, degree of deacetylation > 90%, viscosity 45 mPa s for 1% (w/v) solution, molecular weight: ≈10 kPa, Jinhu Company), glycine (Tokyo Chemical Industry Co., Ltd.), acryloyl chloride (Energy Chemical Reagent Co., Ltd.). Sodium hydroxide (NaOH), hydrochloric acid (HCl), sodium chloride (NaCl), and calcium chloride (CaCl_2_) (Sinopharm Chemical Reagent Co., Ltd.). Iron (III) chloride hexahydrate (FeCl_3_·6H_2_O, J&K Scientific Co., Ltd.), ferric sulfate (Fe_2_(SO_4_)_3_, Aladdin Chemistry Co., Ltd.), aluminium chloride (AlCl_3_, Jinke Fine Chemical Research Institute), magnesium sulfate (MgSO_4_, Macklin Reagent Co., Ltd.), *N*,*N* *′*‐methylene‐bis‐acrylamide (MBA) and 2‐hydroxy‐4*′*‐(2‐hydroxyethoxy)‐2‐methylpropiopheno (Irgacure 2959) (Alfa Aesar Co., Ltd.), tetrahydrofuran, ethyl acetate, and petroleum ether were obtained from Concord Reagent Co., Ltd. and used directly.

### Synthesis of N‐Acryloyl 2‐Glycine (ACG)

ACG was synthesized according to the previously reported method.^[^ ^51^ ^]^ In a 1000 mL two‐necked round‐bottom flask, glycine (45.04 g) was dissolved in water (480 mL) and cooled down with an ice bath. Sodium hydroxide (26.40 g) was added under vigorous stirring. A solution of acryloyl chloride (53.70 mL) in THF (50 mL) was slowly injected into the flask. The pH was maintained at 7.5–7.8. After stirring for 8 h, the reaction mixture was extracted with ethyl acetate. The retained aqueous phase was acidified to pH ≈ 2.0 with 6 mol L^−1^ HCl and then extracted again with ethyl acetate. The organic phase was dried with anhydrous MgSO_4_ overnight. After filtration, it was concentrated by evaporation under reduced pressure and then precipitated in an excess of petroleum ether. A white solid was obtained in 40% yield (31.09 g). ^1^H NMR (400 MHz, DMSO‐d_6_, *δ*): 12.56 (s, 1H), 8.41 (t, 1H), 6.25‐6.32 (m, 1H), 6.07‐6.12 (dd, 1H), 5.60‐5.63 (dd, 1H), 3.82‐3.84 (d, 2H); ^13^C NMR (400 MHz, DMSO‐d_6_, *δ*): 171.33, 165.07, 131.44, 125.80, 40.80.

### Preparation of CS/PACG Composite Hydrogels and DN Hydrogels

The CS/PACG composite hydrogels were prepared by photo‐initiated radical polymerization. Briefly, ACG (0.60 g), CS (0.04, 0.07, and 0.19 g), MBA solution (21.49 µL, 0.03 mol% of ACG monomers, *C*_MBA_ = 10 mg mL^−1^) and Irgacure 2959 (0.01 g, 1 mol% of ACG monomers) were dissolved in deionized water (4 mL). The mixture was cast into desired molds and radiated under UV radiation (150 W) for 2 h. Subsequently, the CS_1.6_/PACG composite hydrogel were soaked in different salt solutions for a predetermined time to prepare various CS/PACG DN hydrogels.

### Structural Characterizations

^1^H NMR and ^13^C NMR spectra were measured on a Bruker Avance 400 or Bruker Avance III 400 HD NMR spectrometer using DMSO‐d_6_ as solvent. Fourier Transform infrared (FTIR) spectra were taken on a TENSOR‐27 spectrometer. The X‐ray diffraction (XRD) patterns were recorded by an Empyrean diffractometer with a scan rate of 4° min^−1^ and scan range from 3–80°. Field emission scanning electron microscopy (SEM) images were acquired from a JSM‐6700F microscope.

### Swelling Ratio of the Hydrogels

The swelling ratio (SR) was calculated as follows

(1) $$\mathrm{SR}=\frac{W_{s}-W_{o}}{W_{o}}$$

where *W*_s_ and *W*_o_ are the weight of the swollen hydrogel and the original hydrogel, respectively. The hydrogels were prepared in plastic tubular molds and then cut into cylindrical samples (diameter 5 mm, thickness 2 mm). Samples were immersed in deionized water at room temperature until swelling equilibrium was reached.

### Dehydration Ratio of the Hydrogels

The dehydration ratio (DR) was calculated as follows

(2) $$\mathrm{DR}=\frac{W_{d}-W_{o}}{W_{o}}$$

where *W*_d_ and *W*_o_ are the weight of the dehydrated hydrogel and the original hydrogel, respectively. The hydrogels were prepared in glass sample bottles and then placed in an open or airtight centrifugal tube at room temperature.

### Mechanical Characterizations

All mechanical tests were measured on an Instron 3365 machine at room temperature. The hydrogels were prepared in plastic tubular molds (diameter 6 mm) for tensile tests and in glass sample bottles (diameter 9 mm) for compressive tests. Note: The diameter of each sample was subject to the actual measurement. Tensile rate: 50 mm min^−1^, compressive rate: 5 mm min^−1^.

## Conflict of Interest

The authors declare no conflict of interest.

## Author Contributions

X.D., X.W., and D.W. conceived the experiments. X.D. performed the experiments and wrote the original draft. H.W. assisted with the experimental procedures. X.D., F.Y., H.S., X.W., and D.W. undertook the data analysis. X.W. and D.W. reviewed the manuscript. All authors have given approval to the final version of the manuscript.

## Supporting information

Supporting Information

## Data Availability

The data that support the findings of this study are available from the corresponding author upon reasonable request.
