# Supplementary material for: One‐Step Soaking Strategy toward Anti‐Swelling Hydrogels with a Stiff “Armor”
Source: Adv Sci (Weinh). 2023 Jan 22;10(9):2206242. doi: 10.1002/advs.202206242 (PMC10037974; doi:10.1002/advs.202206242)
Supplement: Supplementary file 1 — Supporting Information [file ADVS-10-2206242-s001.pdf]

Supporting Information

One-Step Soaking Strategy toward Anti-Swelling Hydrogels with a Stiff "Armor"

Xueyu Dou, Hufei Wang, Fei Yang, Hong Shen, Xing Wang, and Decheng Wu**

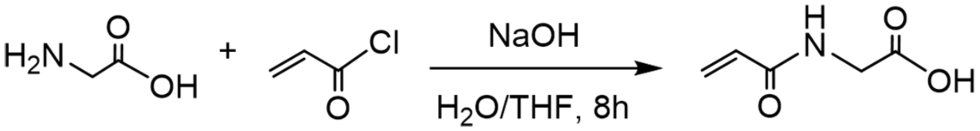

Scheme S1. Synthetic route to *N*-acryloyl 2-glycine (ACG).

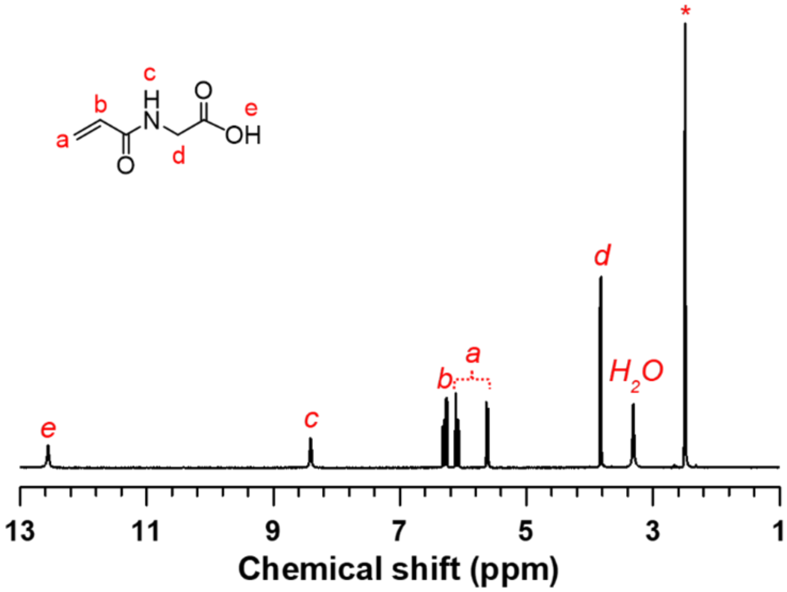

Figure S1. ^{1}H NMR spectrum of ACG at DMSO-d_6.

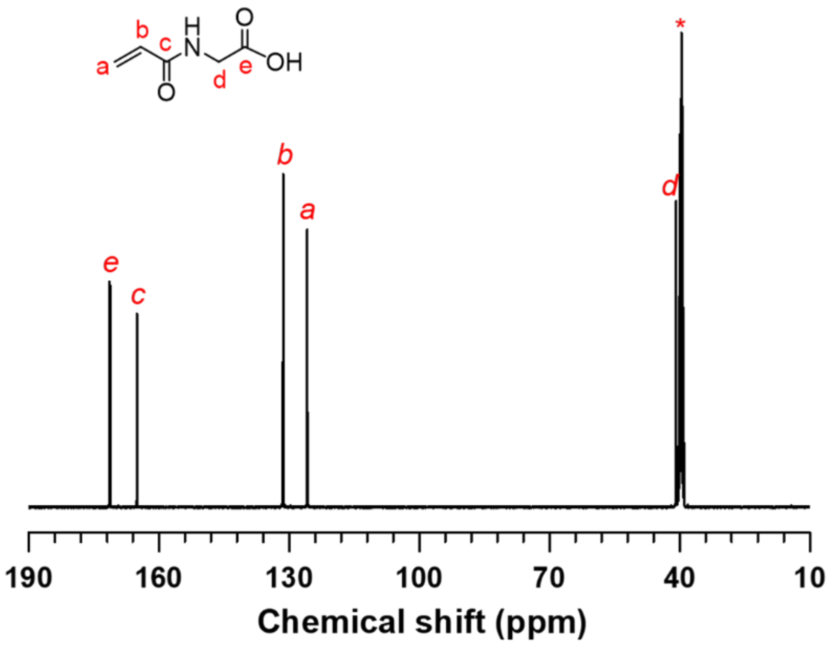

Figure S2. ^{13}C NMR spectrum of ACG at DMSO-d_6.

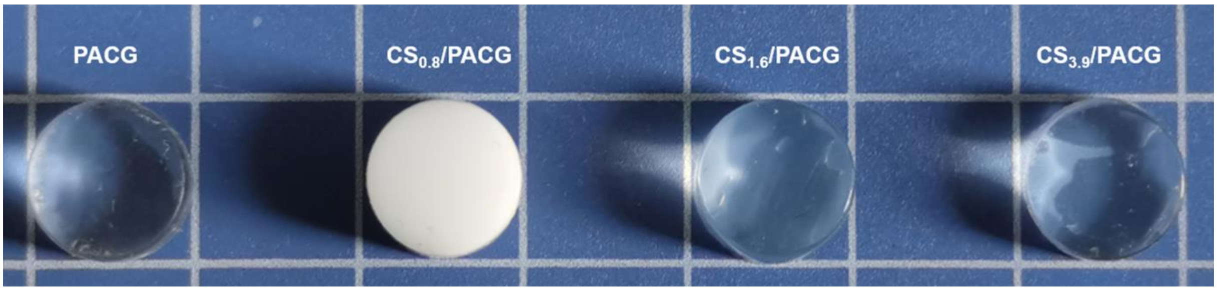

Figure S3. Photographs of CS/PACG composite hydrogels with different contents of CS.

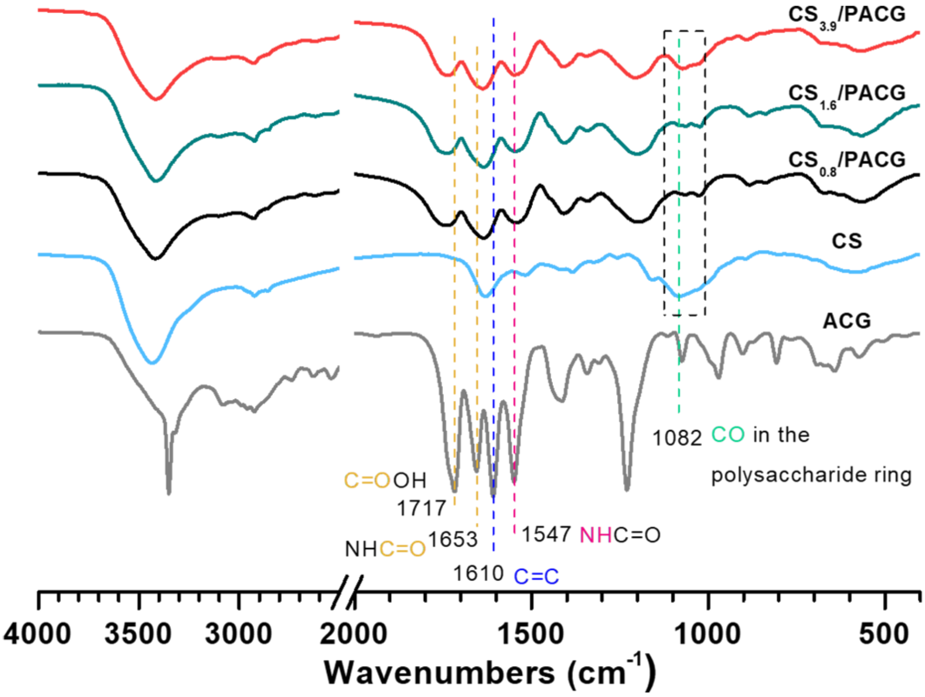

Figure S4. FTIR spectra of ACG, CS and CS/PACG composite hydrogels with different contents of CS.

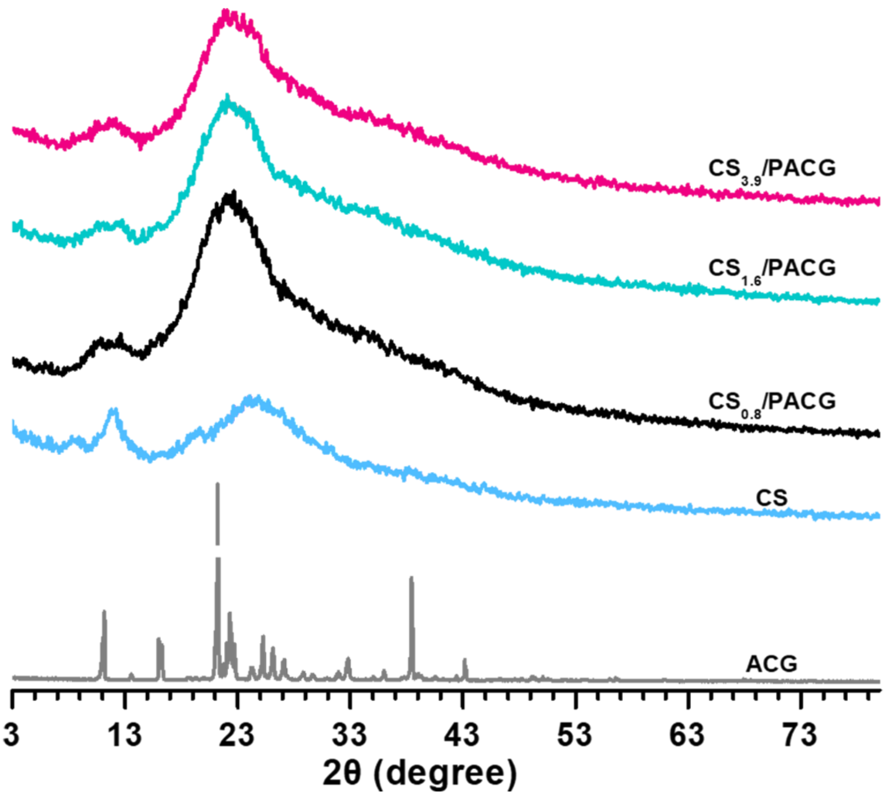

Figure S5. XRD spectra of ACG, CS and CS/PACG composite hydrogels with different contents of CS.

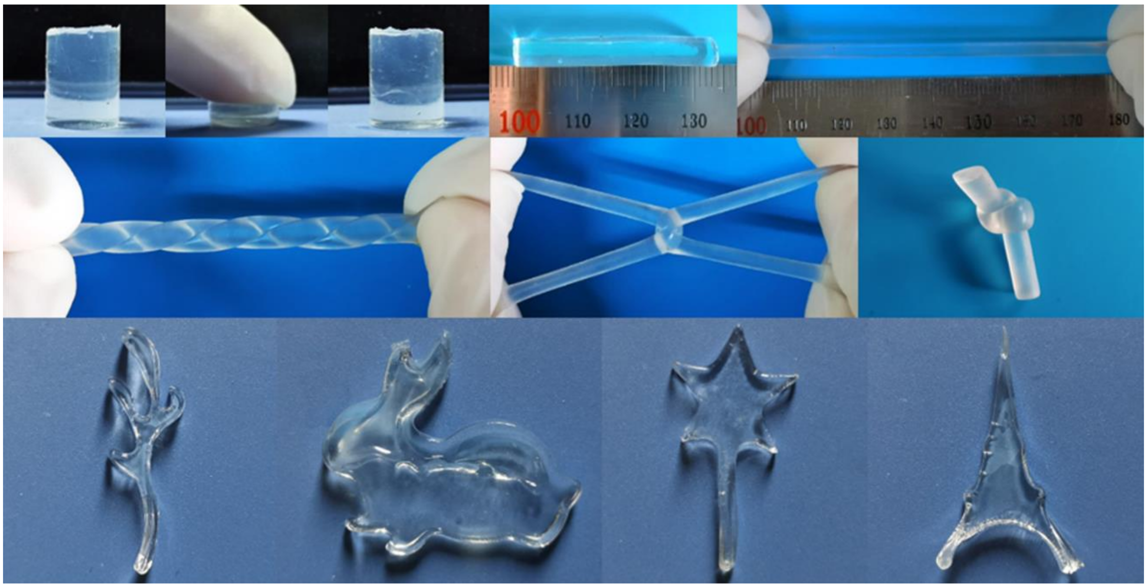

Figure S6. The extraordinary mechanical performance of the $CS_{1.6}$/PACG composite hydrogel: compression, stretching, twisting, crossover stretching, knotting and photographs of the hydrogel with different shapes: the tree branches, rabbit, starlight and stick tower.

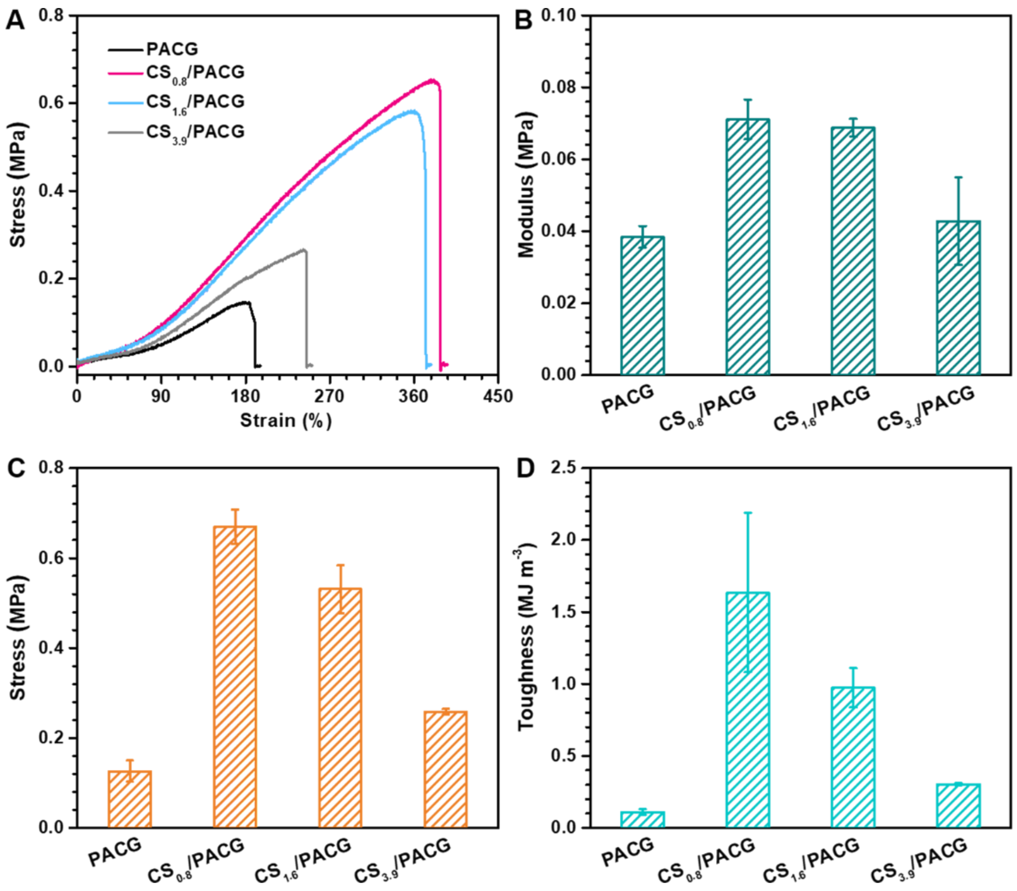

Figure S7. (A) Tensile stress-strain curves, (B) elastic modulus, (C) tensile strength and (D) toughness of the CS/PACG composite hydrogels with different contents of CS.

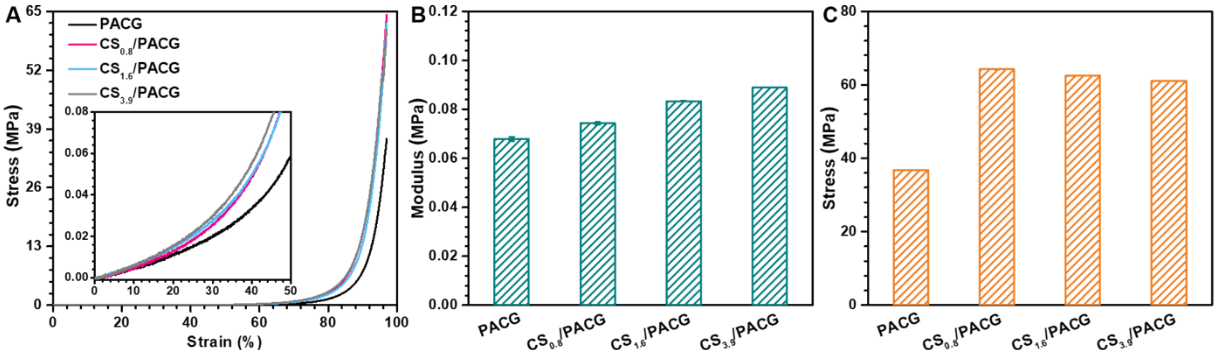

Figure S8. (A) Compressive stress-strain curves, (B) compressive modulus and (C) compressive strength of the CS/PACG composite hydrogels with different contents of CS.

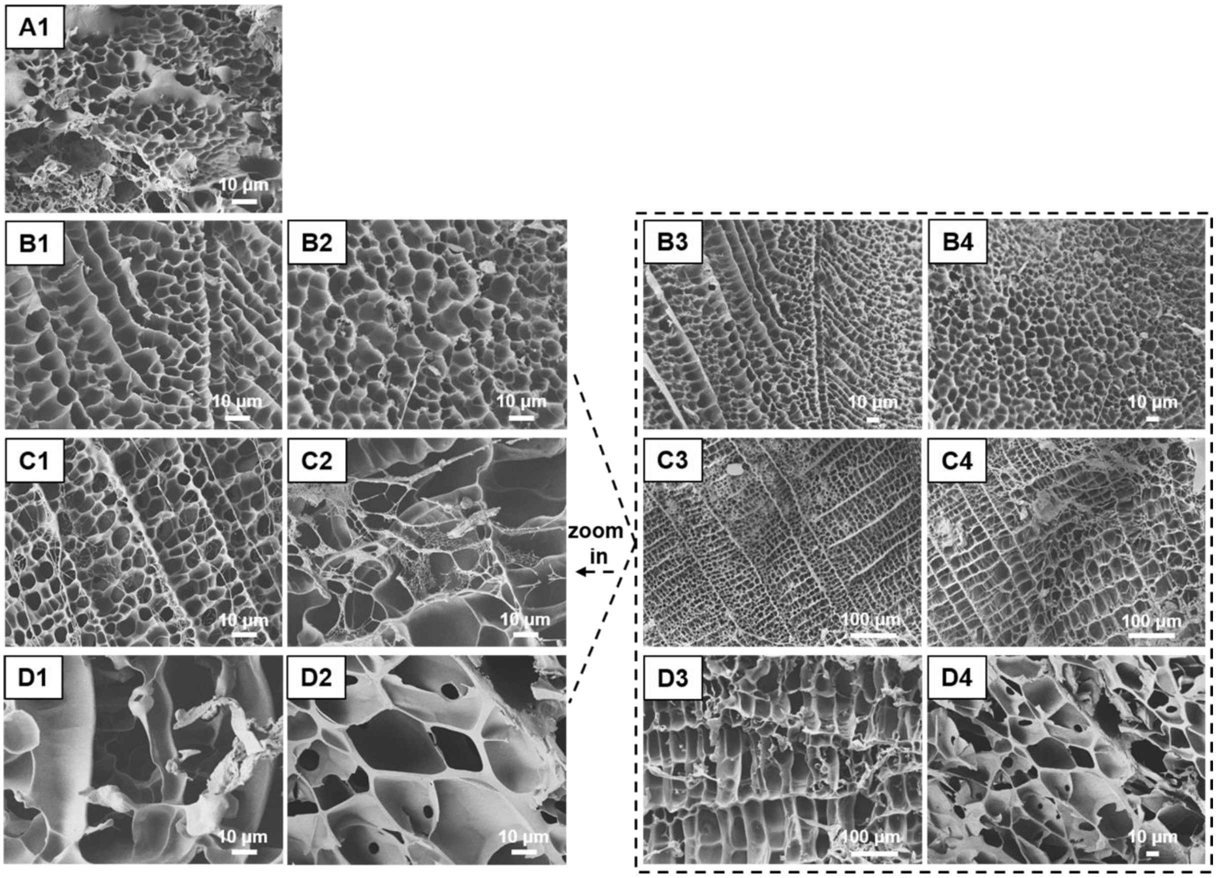

Figure S9. SEM images of the (A1) PACG hydrogel, (B1-B4) $CS_{0.8}$/PACG, (C1-C4) $CS_{1.6}$/PACG and (D1-D4) $CS_{3.9}$/PACG composite hydrogels.

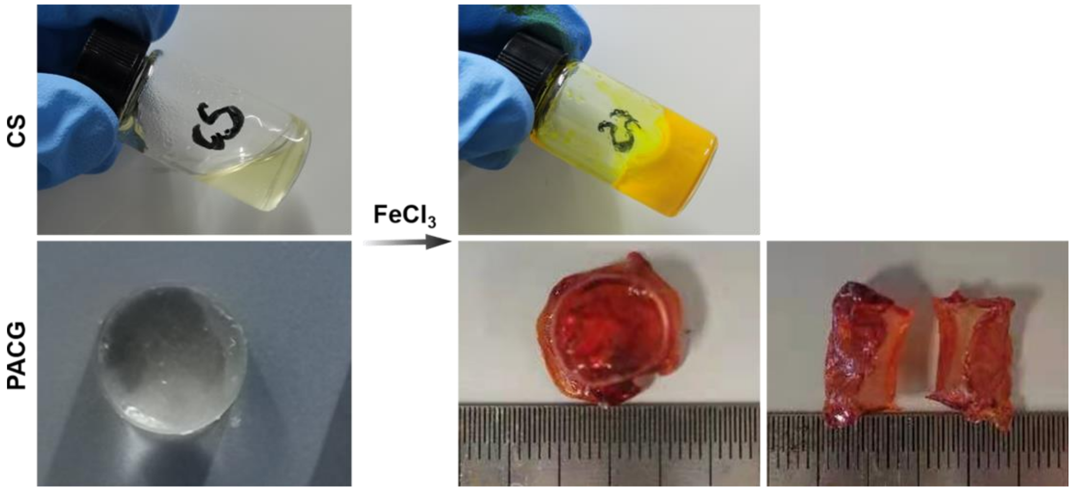

Figure S10. Preparation of the CS chain-entanglement hydrogel and PACG singel network hydrogel treated with $FeCl_3$ solution.

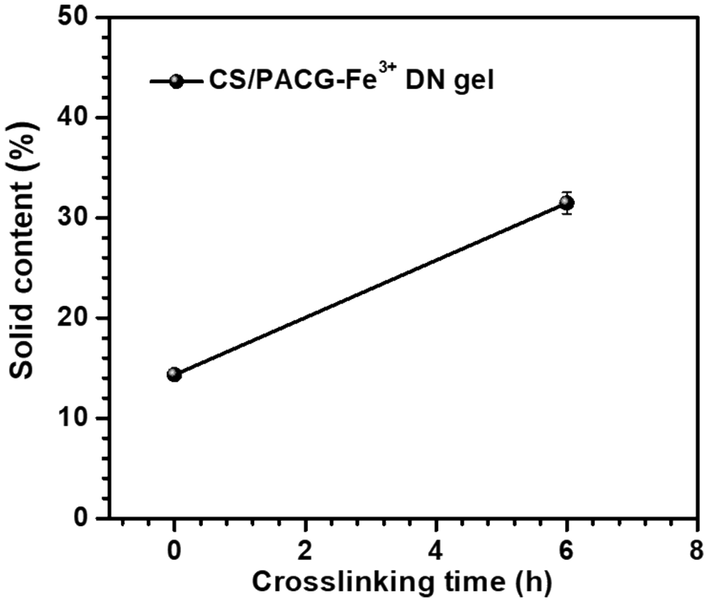

Figure S11. Solid content of hydrogels soaked in 1.5 M $FeCl_3$ solution versus post-crosslinking times.

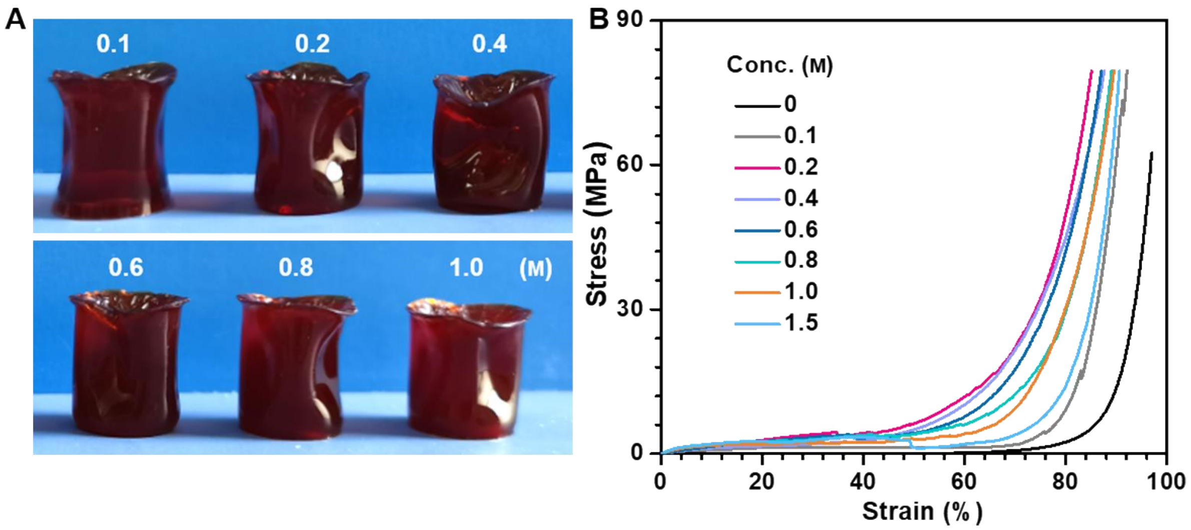

Figure S12. (A) Photographs and (B) compressive stress-strain curves of the CS/PACG-Fe^{3+} DN hydrogels treated with different $FeCl_3$ concentrations (soaking time: 6 h).

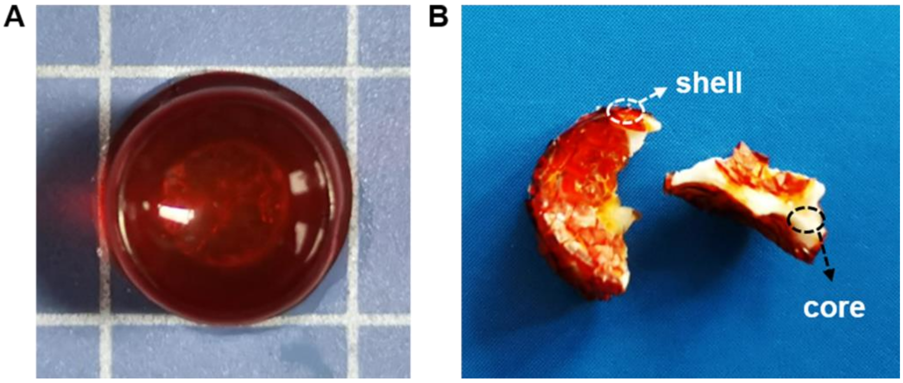

Figure S13. Photographs showing (A) CS/PACG-Fe^{3+} DN hydrogel and (B) its core/shell structure.

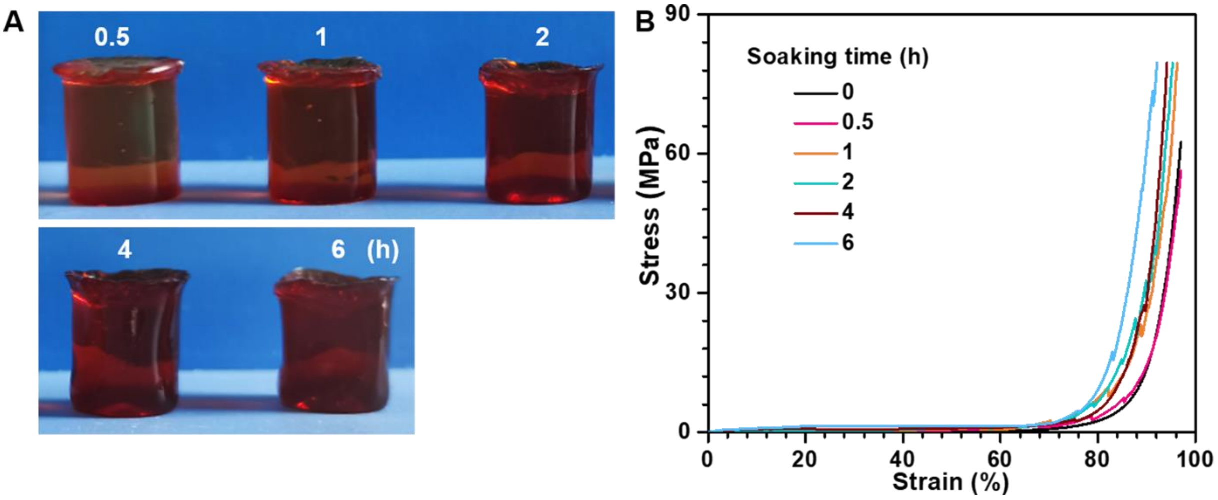

Figure S14. (A) Photographs and (B) compressive stress-strain curves of the CS/PACG-Fe^{3+} DN hydrogels treated with different soaking time ($FeCl_3$ concentration = 0.1 M)

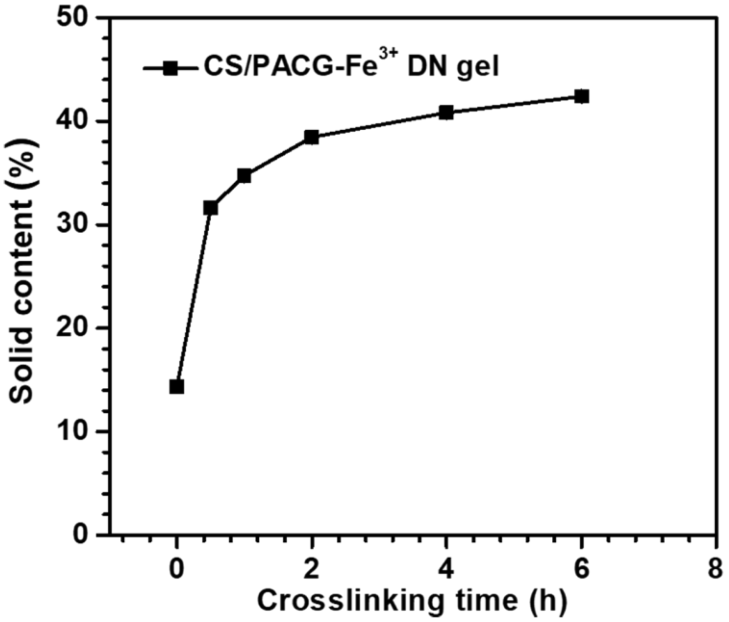

Figure S15. Solid content of hydrogels soaked in 0.1 M $FeCl_3$ solution versus post-crosslinking times.

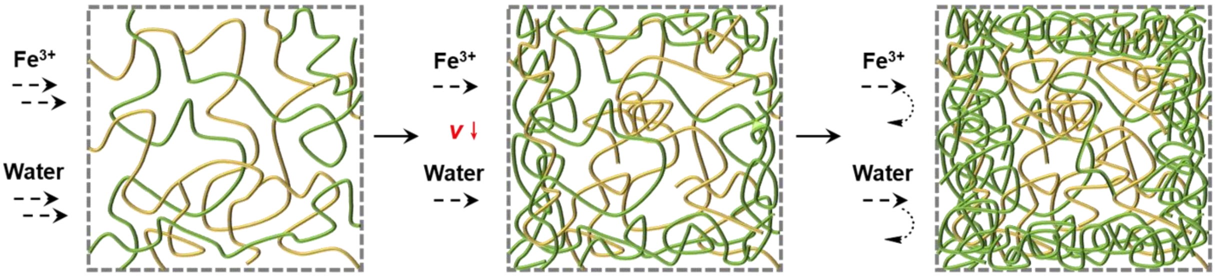

Figure S16. The carboxy-Fe^{3+} tridentate coordination in PACG ionic network induces formation of a dense layer.

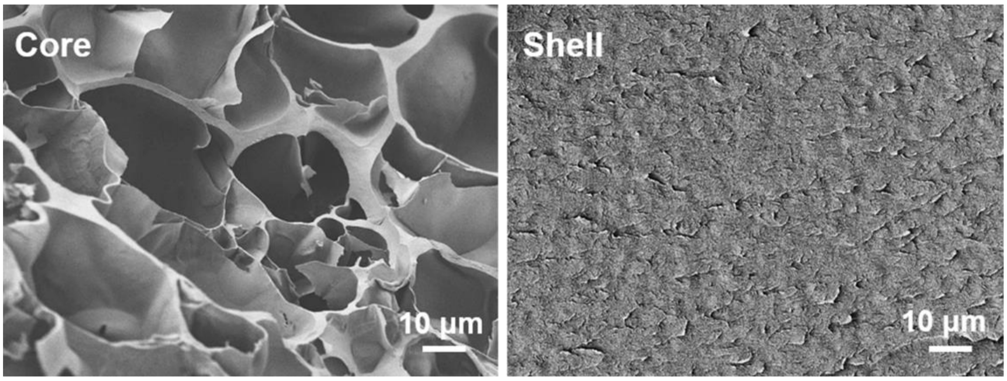

Figure S17. SEM images showing the core/shell structure of CS/PACG hydrogels after soaking in 0.05 M $Fe_2(SO_4)_3$ solution for 1 h.

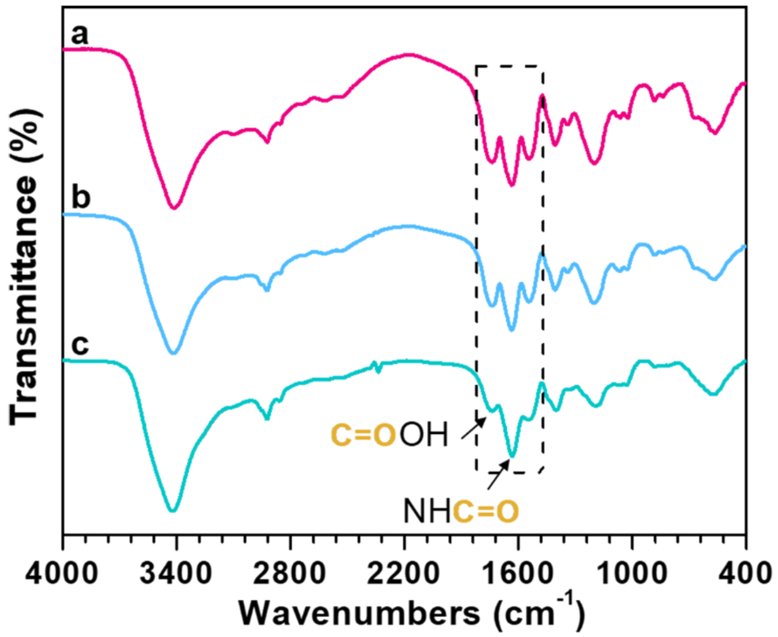

Figure S18. FTIR spectra of the (a) composite hydrogel, (b) core and (c) shell of CS/PACG-Fe^{3+} DN hydrogel.

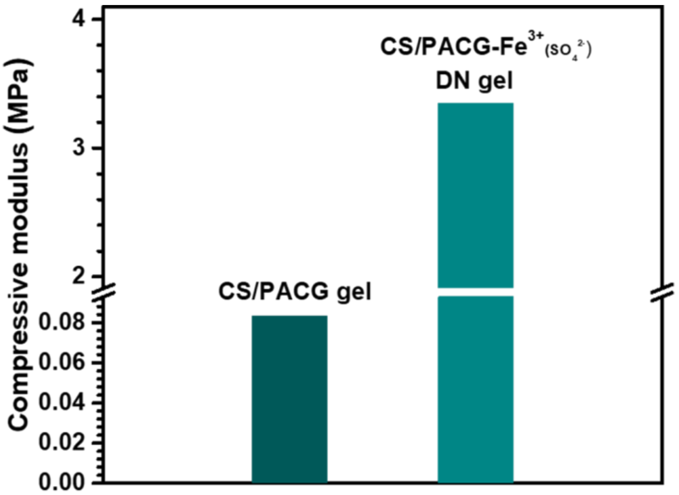

Figure S19. Compressive modulus of the CS/PACG composite hydrogel before and after soaking in $Fe_2(SO_4)_3$ solutions.

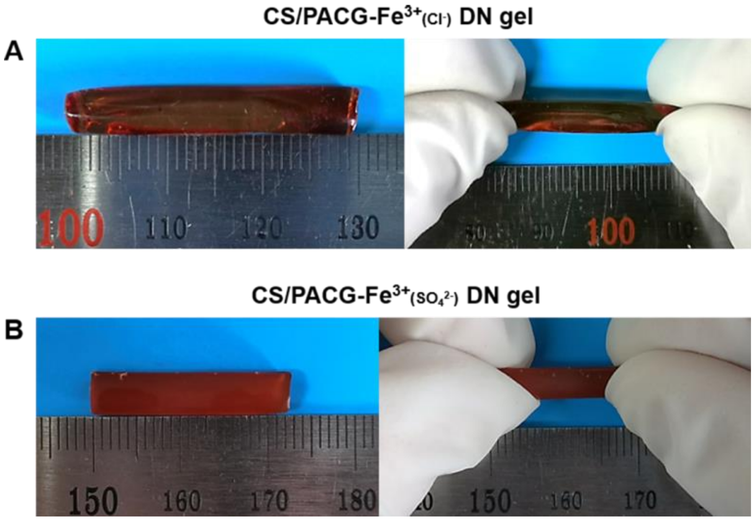

Figure S20. (A) CS/PACG-$Fe^{3+}$$_{(Cl^-)}$ and (B) CS/PACG-$Fe^{3+}$$_{(SO_4^{2-})}$ DN hydrogels with a hard shell layer is not stretchable.

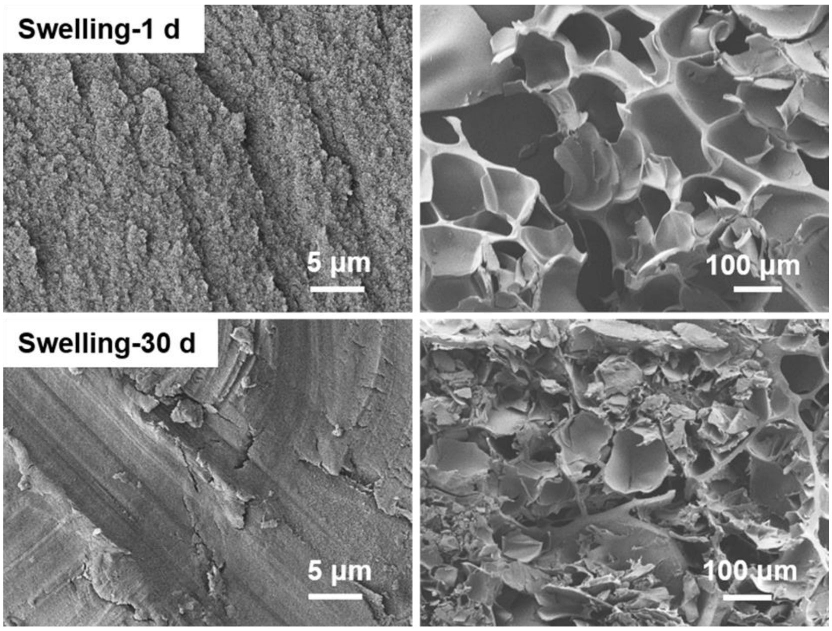

Figure S21. SEM images of the CS/PACG-$Fe^{3+}{}_{(Cl^-)}$ DN hydrogel after 1 and 30 d of swelling.

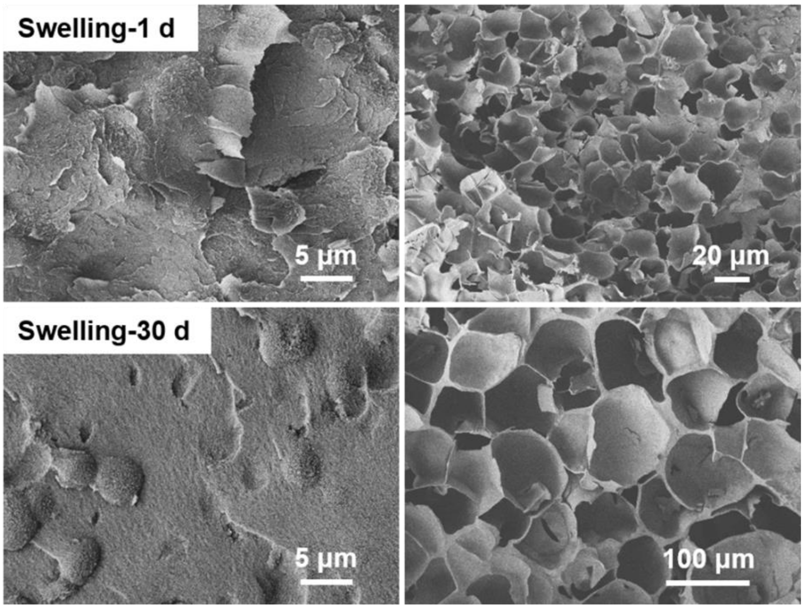

Figure S22. SEM images of the CS/PACG-$Fe^{3+}{}_{(SO_4^{2-})}$ DN hydrogel after 1 and 30 d of swelling.

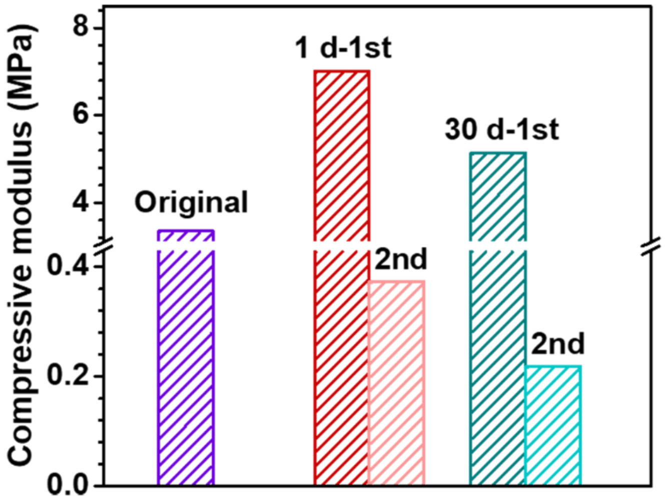

Figure S23. Compressive modulus of the CS/PACG-$Fe^{3+}$$(SO_4^{2-})$ DN hydrogel with different swelling time in water.

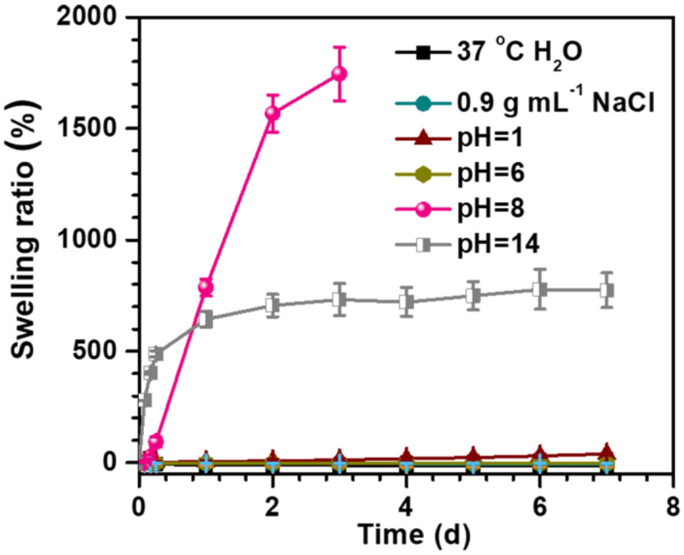

Figure S24. Swelling behavior of the CS/PACG-Fe^{3+} hydrogel in different media.

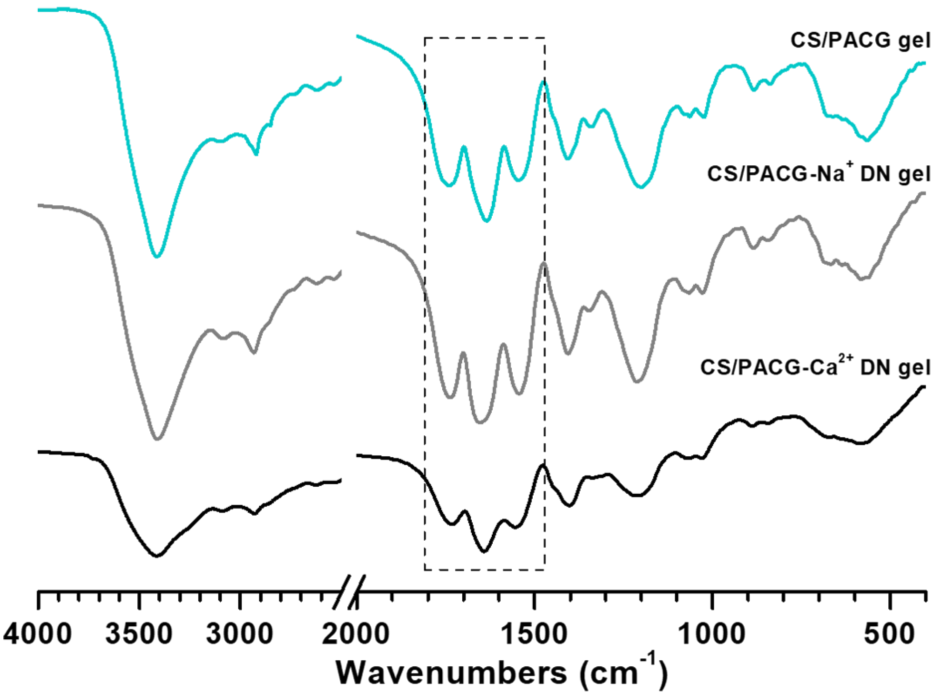

Figure S25. FTIR spectra of the CS/PACG composite hydrogel, CS/PACG-Na^+ and CS/PACG-Ca^{2+} DN hydrogels.

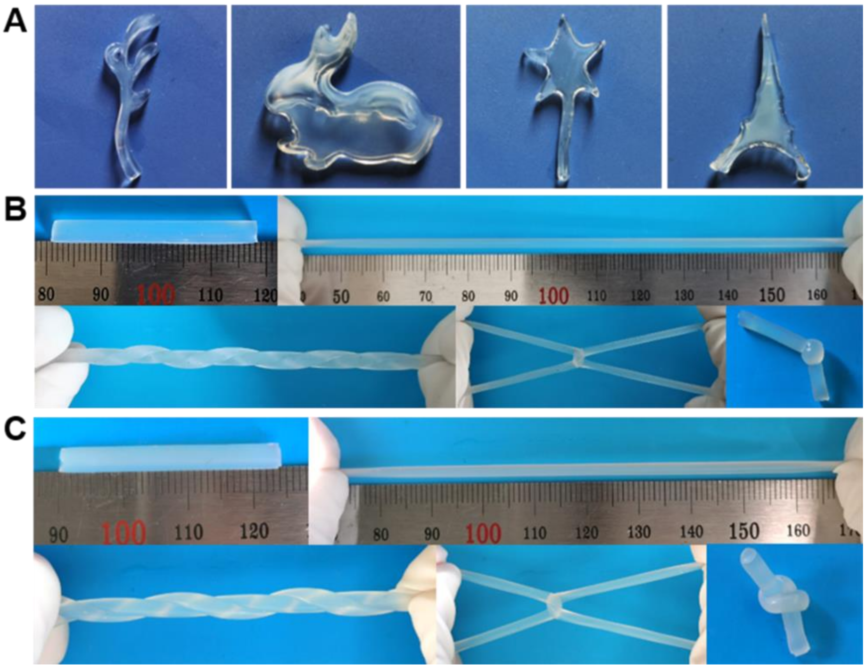

Figure S26. (A) Free-shapeable properties of the CS/PACG DN hydrogels. The extraordinary mechanical properties of (B) CS/PACG-Na^{+} and (C) CS/PACG-Ca^{2+} DN hydrogels: compression, stretching, knotting, twisted stretching and crossover stretching.

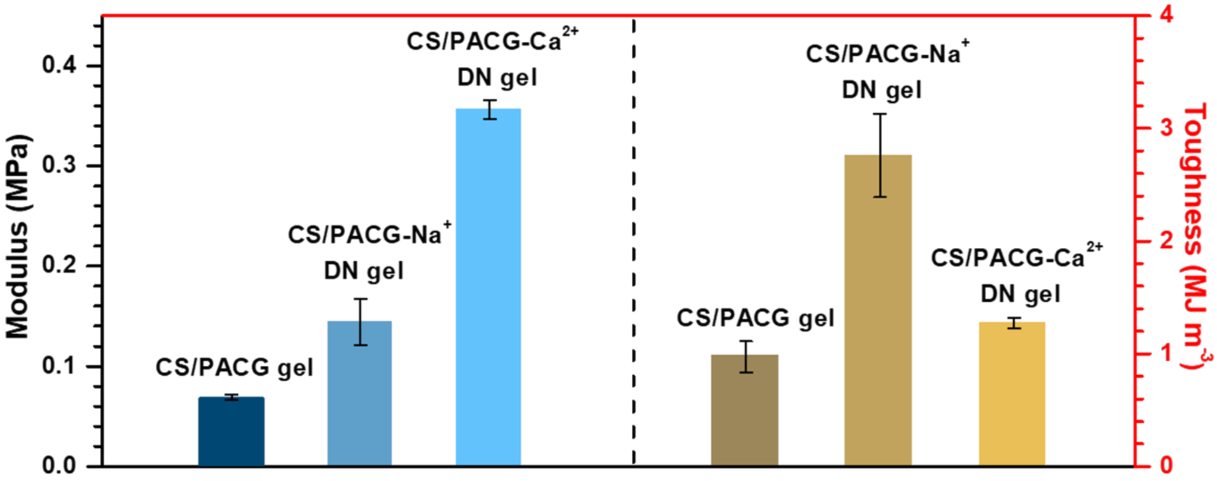

Figure S27. Elastic modulus and toughness of the CS/PACG composite hydrogel before and after soaking in NaCl and $CaCl_2$ solutions.

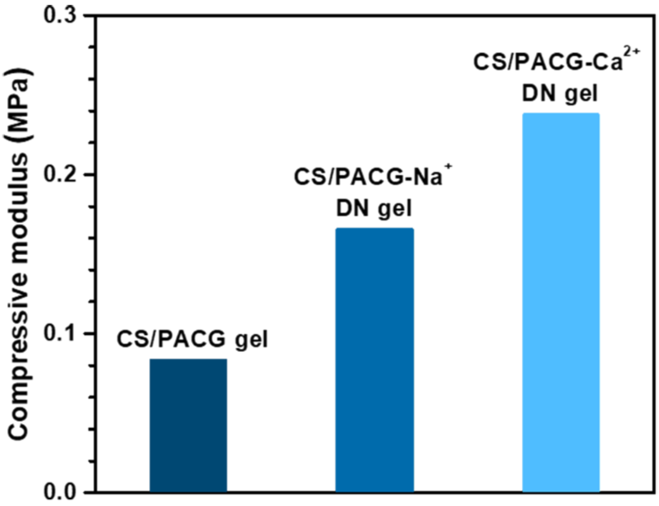

Figure S28. Compressive modulus of the CS/PACG composite hydrogel before and after soaking in NaCl and $CaCl_2$ solutions.

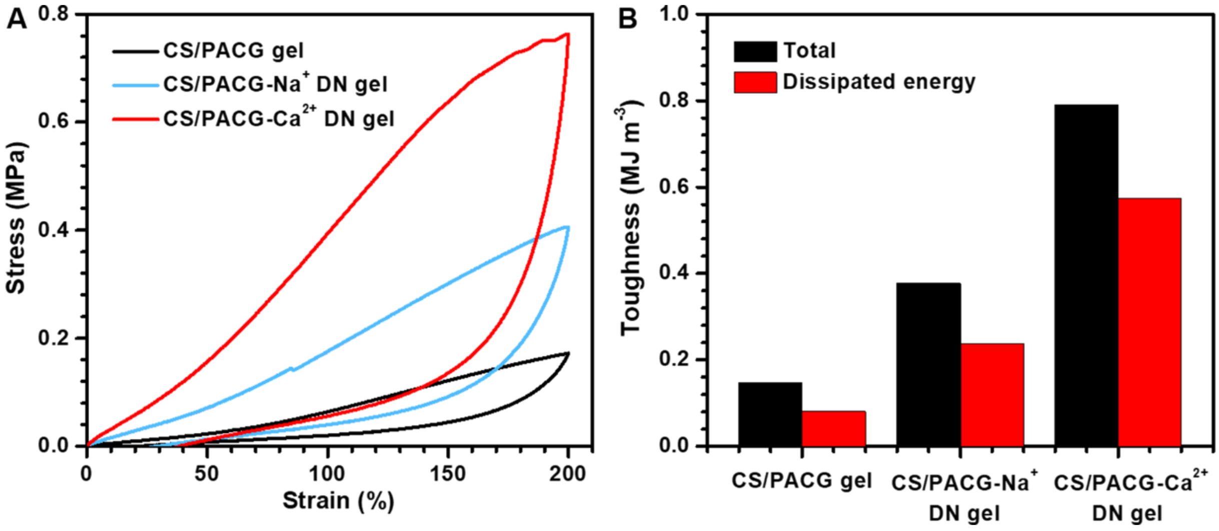

Figure S29. (A) Tensile loading-unloading cycling curves and (B) the corresponding calculated total and dissipated energy (curve area) of the composite hydrogel and CS/PACG-Na^+ and CS/PACG-Ca^{2+} DN hydrogels under the strain of 200%.

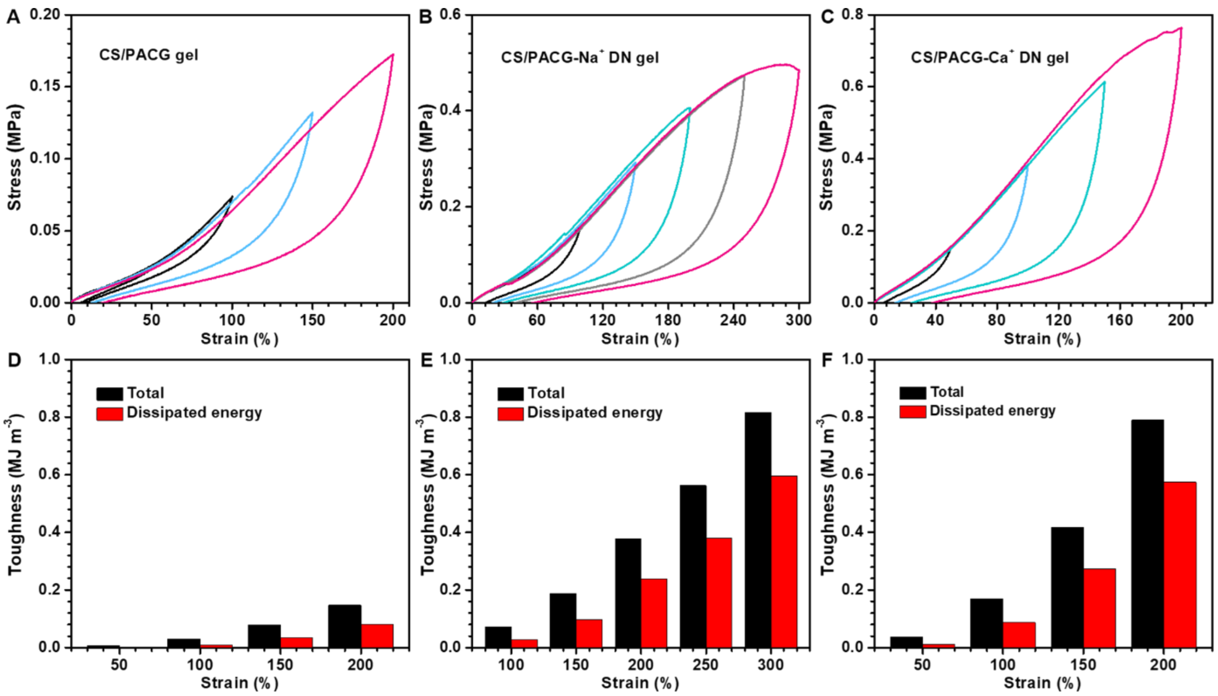

Figure S30. Tensile cyclic loading-unloading curves and the corresponding calculated total and dissipated energy of (A,D) CS/PACG composite hydrogel, (B,E) CS/PACG-Na^{+} and (C,F) CS/PACG-Ca^{2+} DN hydrogels under different strains.

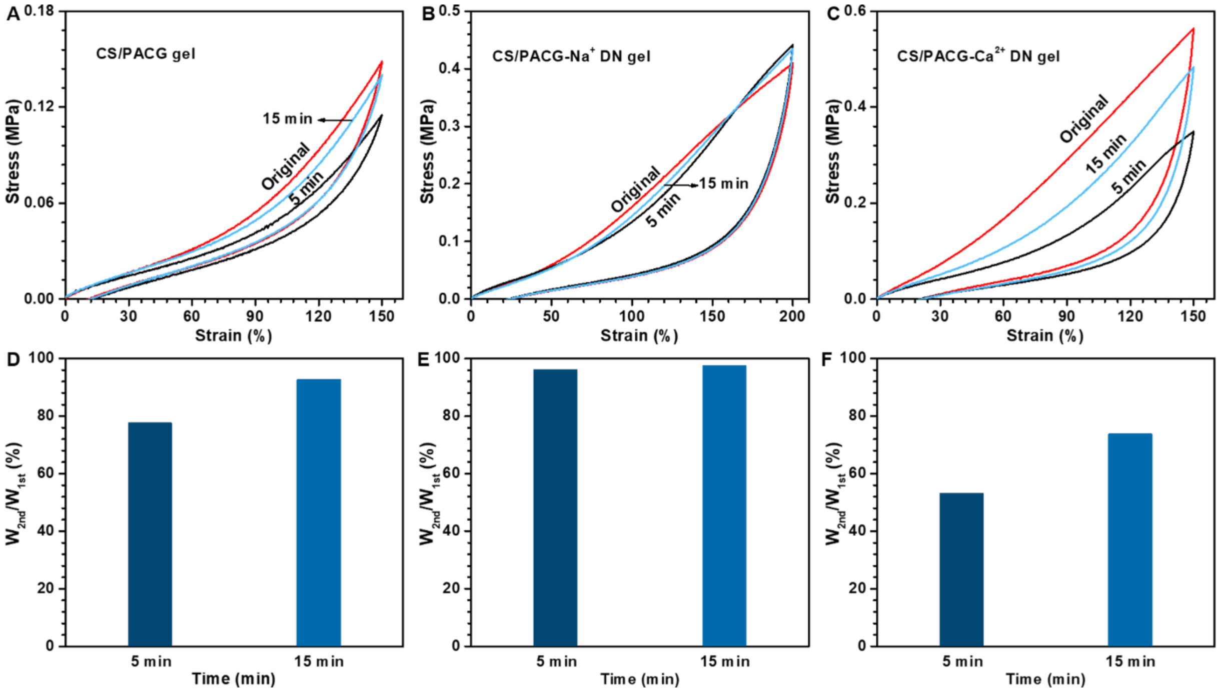

Figure S31. Tensile cyclic loading-unloading curves and recovery efficiency of (A,D) CS/PACG composite hydrogel, (B,E) CS/PACG-Na^{+} and (C,F) CS/PACG-Ca^{2+} DN hydrogels after different resting time at room temperature.

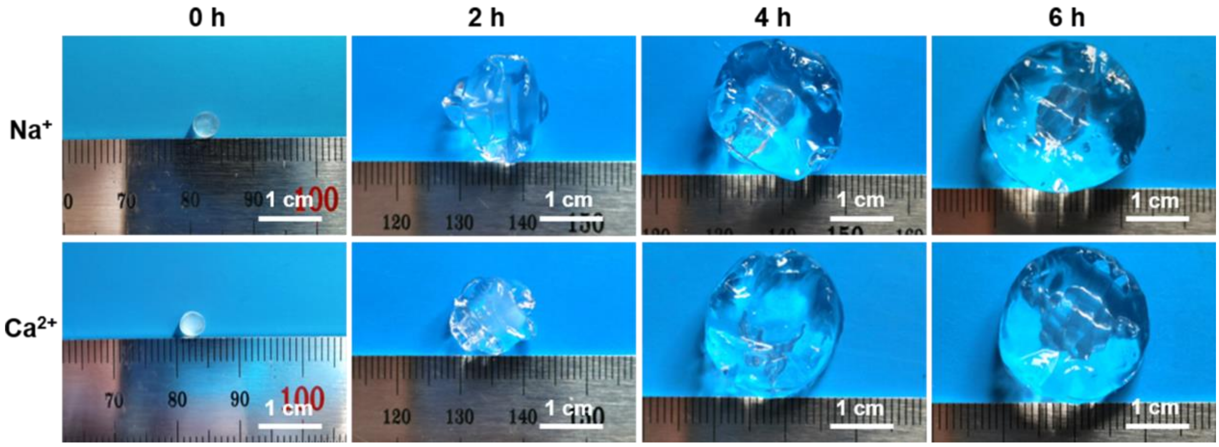

Figure S32. Photographs of the CS/PACG-Na^+ and CS/PACG-Ca^{2+} DN hydrogels after 0, 2, 4 and 6 h of swelling.

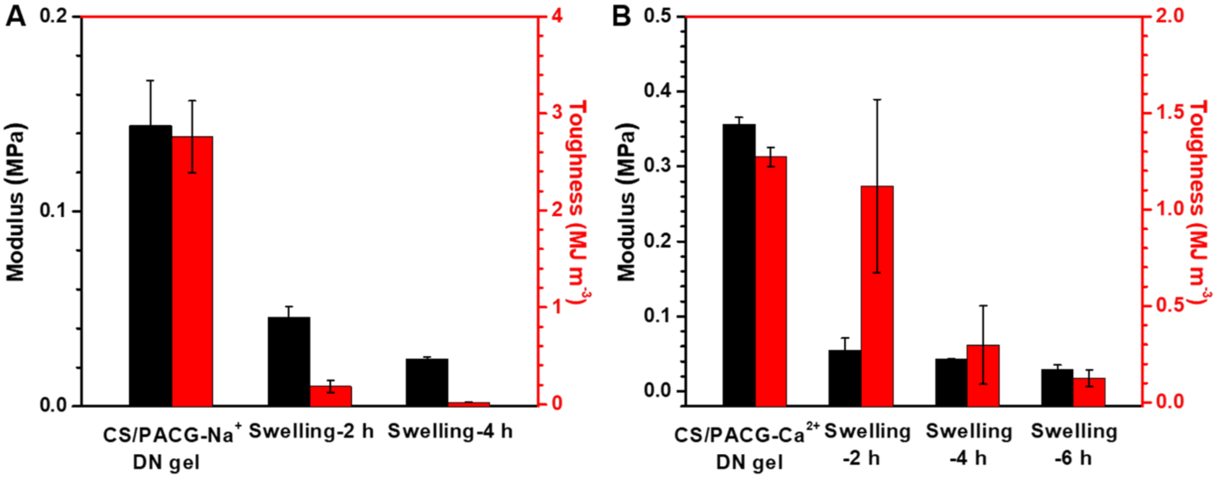

Figure S33. Elastic modulus and toughness of the (A) CS/PACG-Na^+ and (B) CS/PACG-Ca^{2+} DN hydrogels with different swelling time in water.

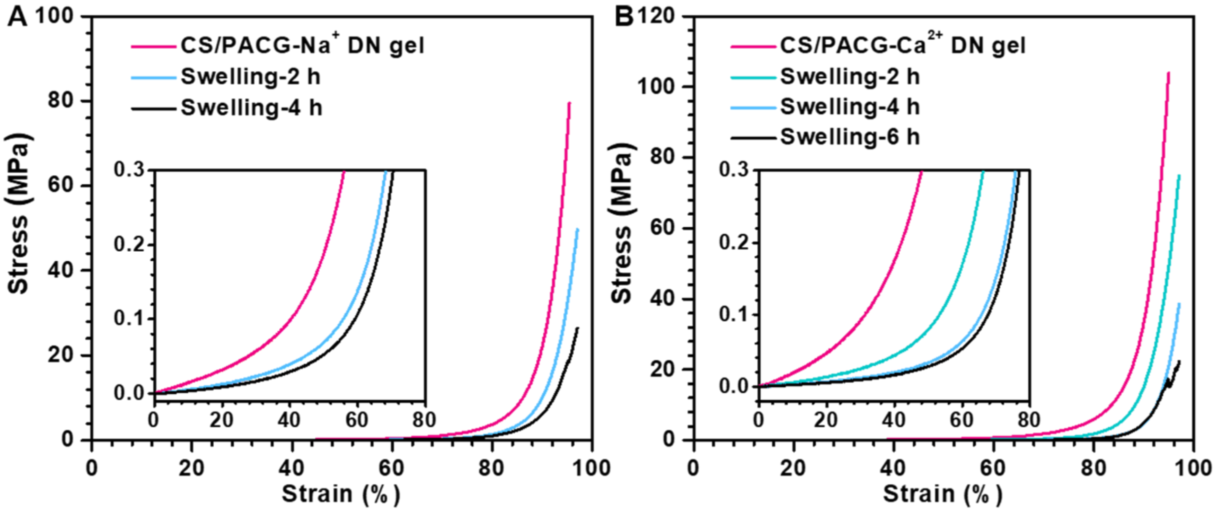

Figure S34. Compressive stress-strain curves of the (A) CS/PACG-Na^+ and (B) CS/PACG-Ca^{2+} DN hydrogels after 0, 2, 4 and 6 h of swelling.

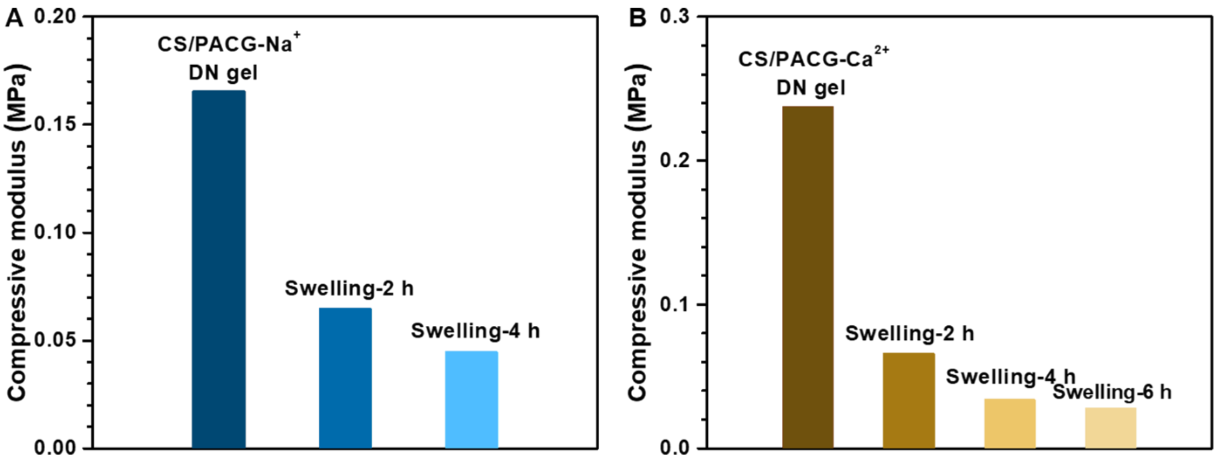

Figure S35. Compressive modulus of the (A) CS/PACG-Na^{+} and (B) CS/PACG-Ca^{2+} DN hydrogels after 0, 2, 4 and 6 h of swelling.

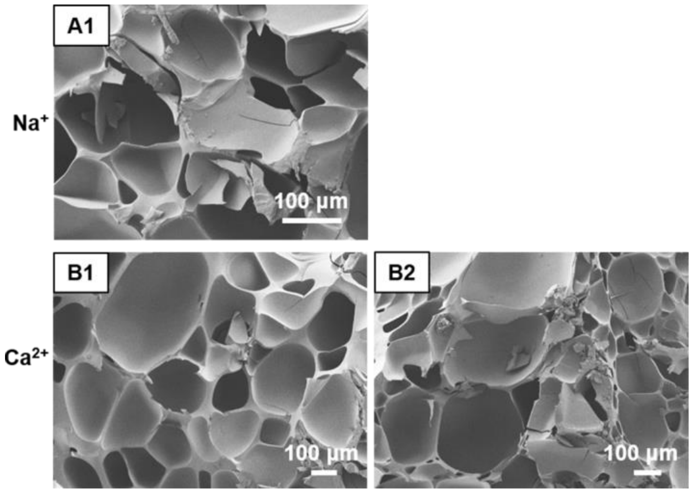

Figure S36. SEM images of (A1) the CS/PACG-Na^{+} and (B1,B2) CS/PACG-Ca^{2+} DN hydrogels after (A1,B1) 2 h and (B2) 4 h of swelling.

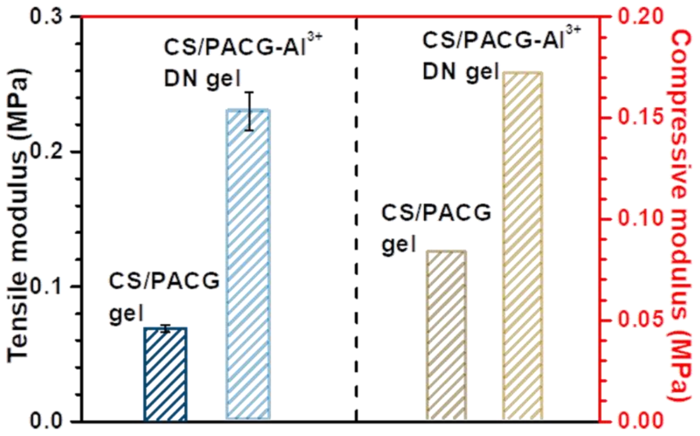

Figure S37. Compressive modulus of the composite hydrogel and CS/PACG-Al^{3+} DN hydrogel.

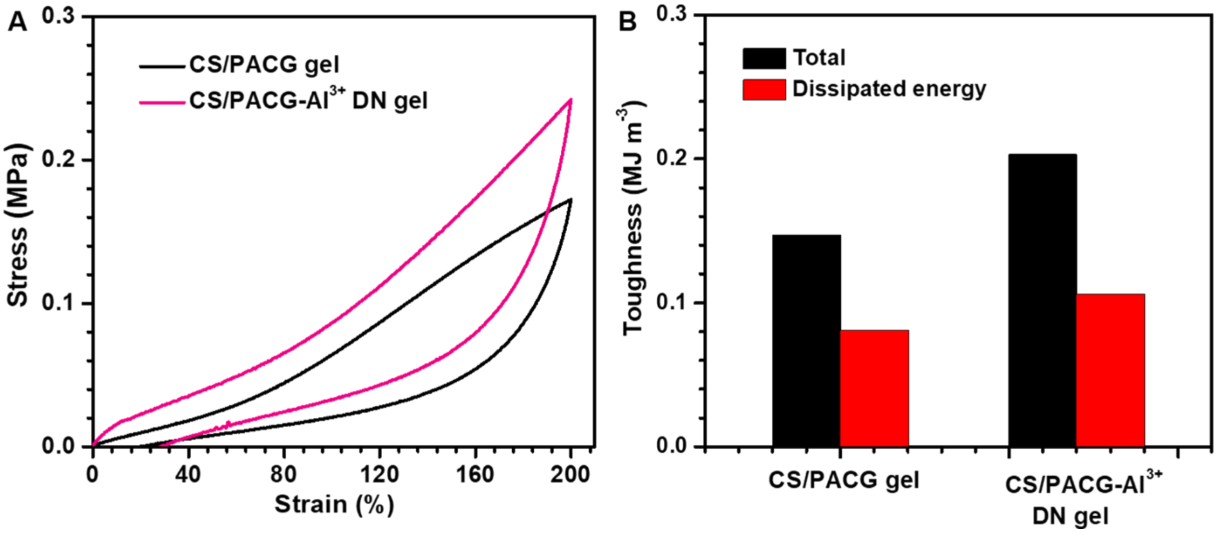

Figure S38. (A) Tensile loading-unloading cycling tests and (B) the calculated total and dissipated energy (curve area) of composite hydrogel and CS/PACG-Al^{3+} DN hydrogel under the strain of 200%.

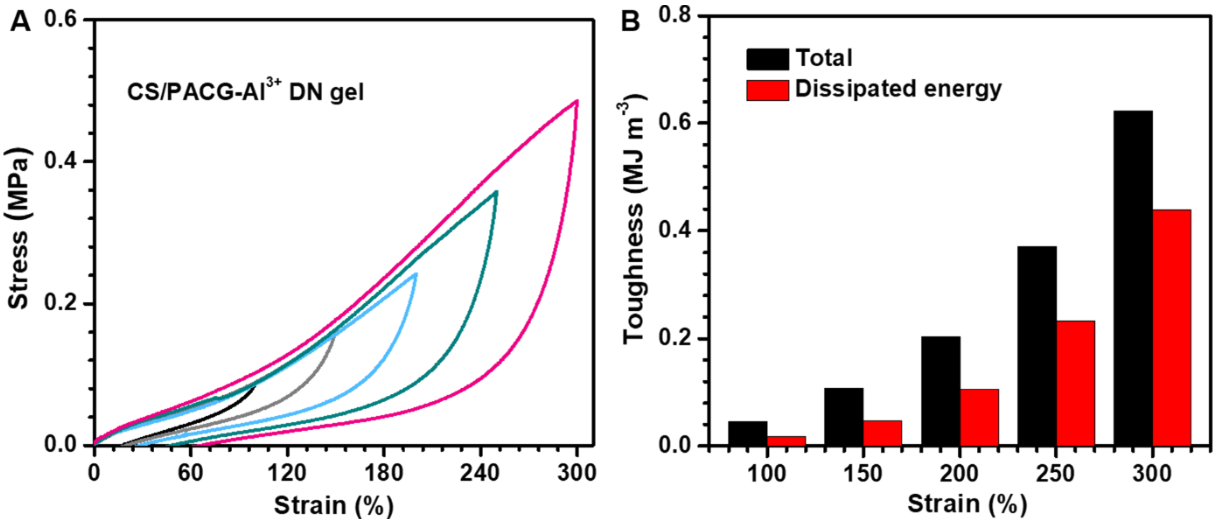

Figure S39. (A) Tensile loading-unloading cycling tests and (B) the calculated total and dissipated energy of CS/PACG-Al^{3+} DN hydrogels under different strains.

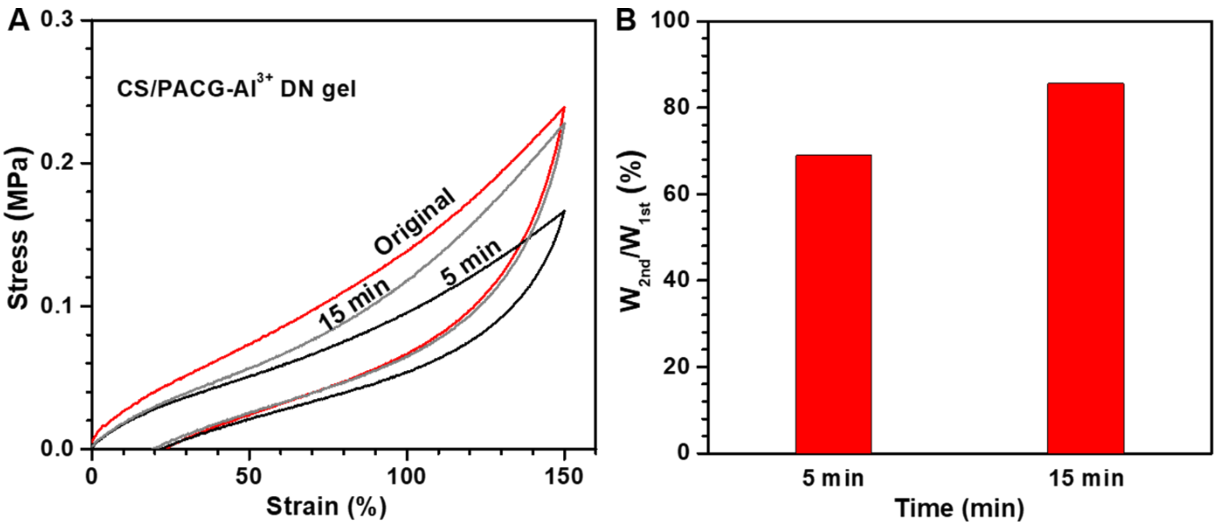

Figure S40. (A) Tensile cyclic loading-unloading curves and (B) recovery efficiency (W_{2nd}/W_{1st}) of CS/PACG-Al^{3+} DN hydrogels after different resting time at room temperature.

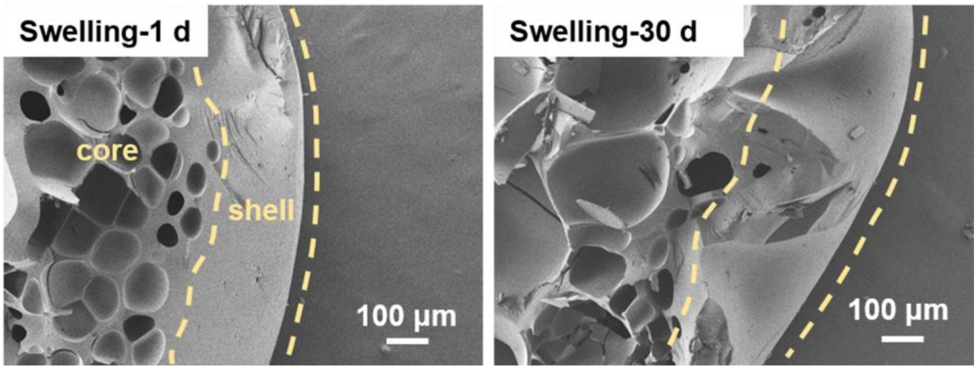

Figure S41. SEM images of the CS/PACG-Al^{3+} DN hydrogels after 1 and 30 d of swelling.

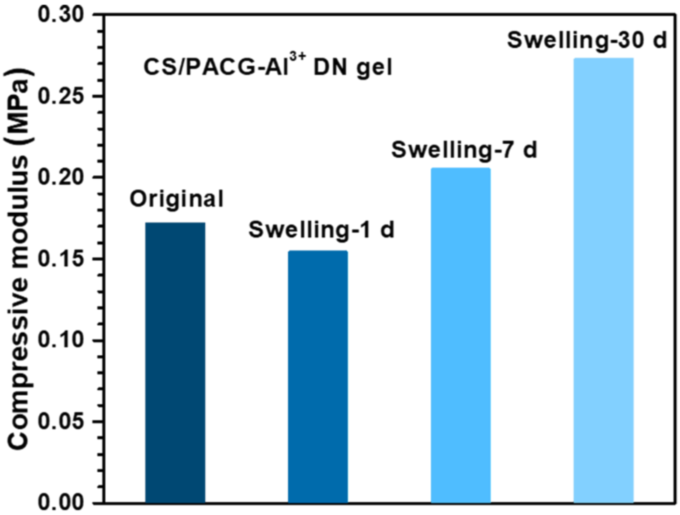

Figure S42. Compressive modulus of the CS/PACG-Al^{3+} DN hydrogels with different swelling time in water.

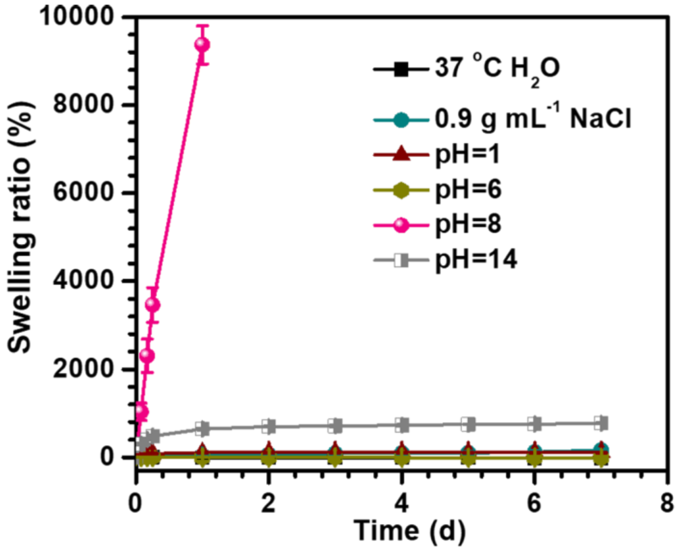

Figure S43. Swelling behavior of the CS/PACG-Al^{3+} hydrogel in different media.
